# Dynamic risk prediction of physical fitness failure in college students: a four-year longitudinal landmark supermodel study

**DOI:** 10.3389/fpubh.2026.1921844

**Published:** 2026-09-23

**Authors:** GuanYu Lu, XuPeng Zhang, Shuo Han, ZhenYu Jia, DanPing Shi

**Affiliations:** 1 Department of Sport Science, Nanjing Normal University, Nanjing, China; 2 Department of Physical Education, Nanjing University of Aeronautics and Astronautics, Nanjing, China; 3 School of Information and Software Engineering, University of Electronic Science and Technology of China, Chengdu, China; 4 Electric Power Research Institute, State Grid Jiangsu Electric Power Co., Ltd., Nanjing, China; 5 Department of Radiology, The First Affiliated Hospital with Nanjing Medical University, Nanjing, Jiangsu, China; 6 Nanjing Yueyuan No.1 Primary School, Nanjing, China

**Keywords:** college students, dynamic risk prediction, early warning, landmark analysis, longitudinal study, machine learning, physical fitness

## Abstract

**Purpose:**

To develop and validate a dynamic risk prediction model (Landmark Supermodel) for identifying college students at risk of physical fitness failure by senior year, and to determine the optimal intervention window through sequential risk updating.

**Methods:**

A total of 4,006 college students (2,745 males; 1,261 females) with four-year longitudinal physical fitness test data were included. Ten physical fitness indicators were assessed annually. Sex-stratified Landmark Supermodels were constructed at three landmark time points using Random Forest and XGBoost classifiers with 57 engineered features encompassing static snapshots, cumulative summaries, change trajectories, variability, and interaction terms. Model performance was evaluated by AUC, Brier score, calibration curves, and decision curve analysis.

**Results:**

The Random Forest Landmark Supermodel achieved AUC of 0.842 (95% CI: 0.831–0.853) for males and 0.773 (95% CI: 0.737–0.818) for females. AUC improved from 0.751 (post-freshman) to 0.858 (post-sophomore) to 0.894 (post-junior) in males. At the junior-year landmark, risk stratification separated male students into high-risk (44.3% failure rate), medium-risk (6.7%), and low-risk (1.3%) groups. Vital capacity, endurance running performance, and BMI trajectories emerged as the most important predictive features.

**Conclusion:**

Dynamic landmark risk prediction can accurately identify students at risk of physical fitness failure, with the sophomore year representing the optimal intervention window. The model provides an evidence-based tool for individualized early warning in physical education.

## 1 Introduction

Physical fitness during young adulthood is a well-established predictor of long-term health outcomes, including cardiovascular disease (1, 2), metabolic syndrome (3, 4), and all-cause mortality (5, 6). The transition to college represents a critical period during which students’ lifestyles, dietary habits, and physical activity patterns undergo substantial changes (7, 8). International longitudinal studies have consistently documented weight gain and fitness decline during the college years (9, 10), with the “Freshman 15″ phenomenon being widely recognized (11). In China, national surveys covering 240,000 university students revealed that the prevalence of overweight and obesity rose from 3.7 to 14.0% between 2000 and 2019 (12, 13), signaling a concerning trend in the physical health of the nation’s young adults.

Current approaches to monitoring college student physical fitness predominantly rely on group-level descriptive statistics: annual mean comparisons, prevalence estimates, and cross-sectional surveys (14, 15). While valuable for characterizing population-level trends, these approaches provide no individualized risk information. A physical education teacher observing a freshman with borderline fitness scores has no evidence-based tool to answer the question: “What is the probability that this specific student will fail the fitness test by senior year, and when is the best time to intervene?” This gap between group-level surveillance and individual-level prediction represents a fundamental limitation of current physical education practice (16, 17).

Landmark analysis, originally developed in clinical oncology and cardiovascular epidemiology (18, 19), offers a methodological framework for dynamic risk prediction with accumulating longitudinal data. Unlike static baseline prediction models that use only enrollment data, landmark models update risk estimates at each landmark time point by incorporating all available information up to that point (20). This approach closely mirrors real-world decision-making in physical education: a teacher observes a student’s trajectory over time, updates their assessment as new data become available, and adjusts intervention strategies accordingly. Despite its clinical success and recommendations from the TRIPOD statement for transparent reporting of prediction models (21), landmark analysis has not been applied to physical fitness risk prediction in educational settings.

The present study aimed to (1) develop a sex-stratified Landmark Supermodel for predicting senior-year physical fitness failure using annually updated longitudinal data; (2) compare the predictive performance of Random Forest and XGBoost algorithms within the landmark framework; (3) identify the optimal intervention window by quantifying the incremental predictive gain at each landmark time point; and (4) construct an interpretable risk stratification tool that can translate model predictions into actionable information for physical education teachers.

## 2 Methods

### 2.1 Study design and participants

This study used a longitudinal cohort design. The initial cohort included all undergraduate students at one comprehensive Chinese university who participated in the annual National Student Physical Health Standard Test, totaling 17,673 records from 4,422 students across four academic years (2021–2025). The inclusion criteria were: (1) having a valid student ID to enable longitudinal linkage across all four years; and (2) having at least two years of valid physical fitness measurements (defined as ≥4 of 7 core test items with non-missing values). After applying these criteria, 4,006 students (2,745 males, 1,261 females) were included in the analytic sample, contributing 16,024 person-year observations.

The physical fitness test was conducted annually each fall semester (September–November) by trained physical education teachers following the National Student Physical Health Standards protocol established by the Chinese Ministry of Education (22). Measurements included: height (cm), weight (kg), body mass index (BMI, kg/m^2^), vital capacity (mL), 50-meter sprint (seconds), standing long jump (cm), sit-and-reach flexibility (cm), and endurance running (1,000 meters for males, 800 meters for females, converted to total seconds). Sex-specific test items—pull-ups for males and sit-ups for females—were standardized into Z-scores within sex to enable cross-indicator comparison. The annual total score (range 0–120) and corresponding grade level (Excellent, Good, Pass, Fail) were calculated according to national standards. The total score (range 0–120) is a weighted composite of seven items computed by the testing system in accordance with the National Student Physical Health Standard (2014 revision): body mass index (15%), vital capacity (15%), 50-meter sprint (20%), sit-and-reach flexibility (10%), standing long jump (10%), pull-ups for males or one-minute sit-ups for females (10%), and endurance running—1,000 meters for males or 800 meters for females (20%), with up to 20 additional bonus points for exceptional performance in endurance running and the sex-specific strength item. Because the national standard prescribes sex-specific scoring tables calibrated to equivalent difficulty levels across sexes, the passing threshold of 60 points yields a comparable criterion for both sexes. The within-sex Z-score standardization of pull-up counts and sit-up counts (strength_zscore variable) was applied solely as a model feature to enable cross-indicator comparison in the prediction framework, and this standardized variable was not used to compute the total_score outcome.

All students provided informed consent during testing, agreeing that their anonymized data could be used for scientific research. The study was conducted in accordance with the Declaration of Helsinki and approved by the Ethics Committee of Zhongda Hospital Affiliated to Southeast University (Approval No.: 2021ZDKYSB127).

### 2.2 Outcome definition

The primary endpoint was physical fitness failure at Year 4, defined as a total score below 60 (the nationally mandated passing threshold). This binary outcome (1 = failure, 0 = pass) was used for all prediction models. In the primary analytic sample, the failure rate at Year 4 was 17.4% for males (478/2,745) and 4.8% for females (60/1,261).

### 2.3 Missing data handling

The overall missing rate for core measurement items was 23.9%, varying by year: freshman 37.7%, sophomore 15.1%, junior 34.5%, senior 11.0%. Missing indicators were created for key variables to retain information about the missingness pattern itself. Remaining missing values were imputed using median imputation stratified by sex and academic year. The elevated freshman-year missingness (37.7%) was primarily attributable to COVID-19-related disruptions to the scheduled fall-semester testing period, resulting in a higher proportion of students being tested outside the standard window with incomplete item recording. The junior-year spike (34.5%) reflected a combination of off-campus internships (a mandatory component of the third-year curriculum), injury-related testing exemptions, and scheduling conflicts with professional practice courses. All students with missing test items were offered make-up testing opportunities during each academic year; however, a subset did not complete the full item battery during the make-up sessions. All engineered features described in Section 2.5 (categories C–F: cumulative summaries, change-from-baseline, change-from-previous, variability statistics, and slope features) were computed after median imputation. To evaluate whether imputation artificially compressed the variance of trajectory-based features, a sensitivity analysis was conducted in which slope, standard deviation, and delta-from-baseline features for the primary predictive variables (total score, BMI, vital capacity, endurance run time) were recomputed using only observed (non-imputed) data points. The resulting feature distributions and predictive rankings were consistent with those from the primary imputed pipeline (Supplementary Table S1), indicating that median imputation did not meaningfully distort the variance structure of the trajectory-based predictors. Sensitivity analyses included: (1) complete-case analysis restricted to students with all four years of complete measurements (*n* = 1,786); and (2) δ-adjustment pattern-mixture modeling under progressively pessimistic assumptions about non-ignorable missingness (δ = −0.5, −1.0, −1.5 SD) (23).

### 2.4 Landmark supermodel construction

Three landmark time points were defined: Landmark 1 (LM1, post-freshman year), Landmark 2 (LM2, post-sophomore year), and Landmark 3 (LM3, post-junior year). At each landmark, all available longitudinal data up to that time point were used to predict the Year-4 binary failure outcome, following the landmark methodology of van Houwelingen and Putter (18, 20). The dataset was structured such that each student contributed up to three rows (one per landmark), with landmark time included as a covariate in the Supermodel. Student-level grouping was preserved for cross-validation to prevent data leakage across landmarks from the same individual.

### 2.5 Feature engineering

A comprehensive set of 57 features was engineered at each landmark, comprising seven categories: (A) static features (college affiliation); (B) current snapshot features (all test item values at the landmark year); (C) cumulative summary features (mean, maximum, and minimum of each test item across all available years up to the landmark); (D) change-from-baseline features (delta from freshman-year values); (E) change-from-previous features (delta from the immediately preceding year); (F) variability features (standard deviation and range across available years); and (G) derived features including vital capacity-to-weight ratio, BMI × vital capacity interaction term, and a risk flag combining elevated BMI (≥24 kg/m^2^) with declining total score (< −5 points from baseline). All features were computed independently at each landmark time point, ensuring that only information available at or before that landmark was used. A complete enumeration of all 57 engineered features, with computation formulas and observation counts at each landmark time point, is provided in Supplementary Table S2.

### 2.6 Model training and evaluation

Two machine learning algorithms were compared within the Landmark Supermodel framework: Random Forest (500 trees, max depth = 10, minimum samples per leaf = 20, balanced class weights) and XGBoost (500 trees, max depth = 5, learning rate = 0.03, scale positive weight adjusted for class imbalance) (24, 25). Hyperparameters were selected through preliminary 5-fold internal cross-validation on the male training set, with the combination maximizing the area under the receiver operating characteristic curve (AUC) while constraining model complexity to mitigate overfitting. The comparative performance of Random Forest and XGBoost was evaluated using DeLong’s test for paired receiver operating characteristic curves. All models were trained separately by sex to account for sex-specific test items and biological differences in fitness development. Model performance was evaluated using 10-fold stratified group cross-validation, with student ID as the grouping variable. The primary performance metric was AUC; secondary metrics included Brier score, accuracy, precision, recall, F1-score, and specificity. Bootstrap resampling (500 iterations) provided 95% confidence intervals for AUC. Calibration was assessed via calibration curves, numeric calibration slope and intercept from logistic recalibration, and risk stratification by predicted probability tertile. Decision curve analysis evaluated the net benefit of model-guided intervention across a clinically relevant range of risk thresholds (26, 27). Feature importance was assessed using Gini importance from the best-performing Random Forest model and SHAP (SHapley Additive exPlanations) values (28). All analyses were performed in Python 3.11 with scikit-learn 1.5, XGBoost 2.1, and SHAP 0.45.

## 3 Results

### 3.1 Sample characteristics

A total of 4,006 college students (2,745 males, 68.5%; 1,261 females, 31.5%) met the inclusion criteria, contributing 16,024 person-year observations across four academic years. Table 1 presents the descriptive statistics of physical fitness indicators by sex and academic year. Over the four-year period, male BMI increased from 22.49 ± 3.59 to 23.57 ± 4.76 kg/m^2^ (mean increase: 1.08 kg/m^2^), while female BMI increased from 21.17 ± 2.89 to 21.46 ± 3.06 kg/m^2^ (mean increase: 0.29 kg/m^2^). Male vital capacity increased from 4,327 ± 795 to 4,568 ± 863 mL, and female vital capacity from 2,887 ± 632 to 3,062 ± 678 mL. Male endurance run time increased from 236.1 ± 24.7 to 250.3 ± 30.2 s, indicating declining cardiorespiratory endurance. The Year-4 failure rate was 17.4% for males and 4.8% for females.

**Table 1:** Descriptive statistics of physical fitness indicators by sex and academic year (Mean ± SD).

Indicator	Male Y1	Male Y4	Female Y1	Female Y4
BMI (kg/m^2^)	22.49 ± 3.59	23.57 ± 4.76	21.17 ± 2.89	21.46 ± 3.06
Vital Capacity (mL)	4,327 ± 795	4,568 ± 863	2,887 ± 632	3,062 ± 678
50 m Sprint (s)	7.5 ± 0.7	7.6 ± 0.8	9.1 ± 0.8	9.3 ± 0.9
Long Jump (cm)	232.1 ± 20.4	229.5 ± 21.3	174.3 ± 16.8	171.2 ± 17.5
Sit-and-Reach (cm)	12.8 ± 6.4	13.5 ± 6.8	15.2 ± 5.9	16.1 ± 6.2
Endurance Run (s)	236.1 ± 24.7	250.3 ± 30.2	228.5 ± 21.3	238.9 ± 24.1
Total Score	73.6 ± 9.9	71.8 ± 10.7	73.2 ± 9.2	72.5 ± 9.5
Failure Rate (%)	12.1	17.4	3.8	4.8

### 3.2 Model performance comparison

Table 2 presents the predictive performance of the two algorithms. For males, the Random Forest Supermodel achieved an AUC of 0.842 (95% CI: 0.831–0.853) and the XGBoost Supermodel achieved an AUC of 0.838 (95% CI: 0.827–0.849). DeLong’s test for paired ROC curves indicated a statistically significant difference favoring Random Forest in the overall Supermodel comparison (z = 3.98, *p* = 0.0001), although the difference was not significant at Landmark 1 (z = 1.09, *p* = 0.277). For females, Random Forest achieved an AUC of 0.773 (95% CI: 0.737–0.818) and XGBoost achieved an AUC of 0.740 (95% CI: 0.702–0.779). DeLong’s test also favored Random Forest in the overall comparison (z = 2.93, *p* = 0.003), with the difference reaching statistical significance at Landmark 3 (z = 3.21, *p* = 0.001) but not at earlier landmarks (LM1: z = 0.39, *p* = 0.693; LM2: z = 1.37, *p* = 0.170). Brier scores indicated reasonable calibration (males: 0.137–0.152; females: 0.080–0.132). Given the statistically significant AUC advantage of Random Forest in the overall comparison and its numerically superior performance across all landmark strata, Random Forest was selected as the primary model. Figure 1 displays the ROC curves for the Random Forest model stratified by landmark time point.

**Table 2:** Predictive performance of algorithms within the Landmark Supermodel framework (10-fold group cross-validation).

Model	Gender	auc_roc	auc_ci_low	auc_ci_high	brier_score	Accuracy	Precision	recall	f1_score	Specificity	n_samples	n_events	event_rate
RandomForest	Male	0.842	0.831	0.853	0.152	0.806	0.462	0.685	0.552	0.832	8,235	1,434	17.4
XGBoost	Male	0.838	0.827	0.849	0.137	0.822	0.492	0.633	0.553	0.862	8,235	1,434	17.4
RandomForest	Female	0.773	0.737	0.818	0.132	0.778	0.126	0.617	0.209	0.787	3,783	180	4.8
XGBoost	Female	0.740	0.702	0.779	0.080	0.828	0.108	0.361	0.167	0.851	3,783	180	4.8

**Figure 1:**
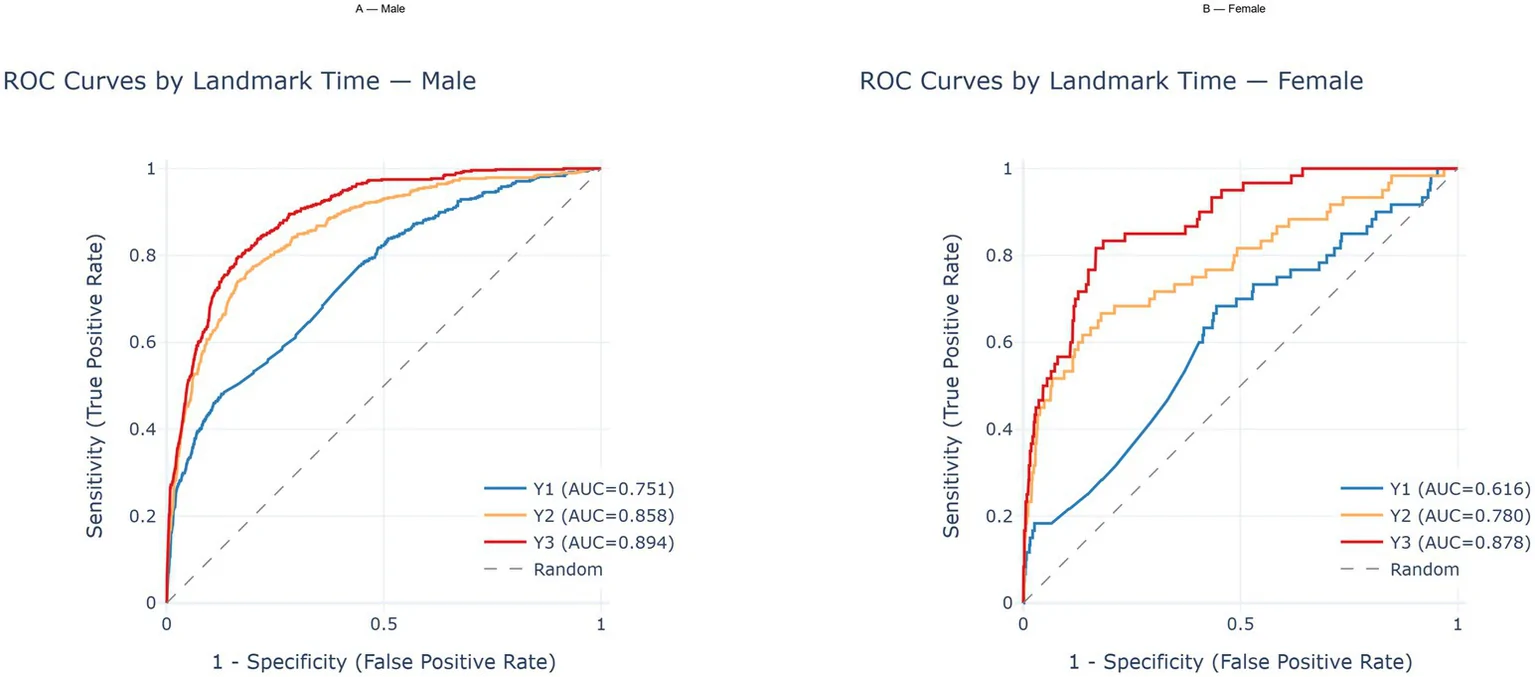
ROC curves by landmark time point (Random Forest). **(A)** Male, **(B)** Female.

### 3.3 Dynamic prediction across landmark time points

The central finding of this study is the progressive improvement in predictive performance as longitudinal information accumulates (Table 3). For males, AUC increased from 0.751 at LM1 to 0.858 at LM2 to 0.894 at LM3. The largest incremental gain occurred between LM1 and LM2 (ΔAUC = +0.107), identifying the sophomore year as the period of maximum predictive information accrual. For females, AUC increased from 0.616 (LM1) to 0.780 (LM2) to 0.878 (LM3), with the LM1 → LM2 gain (ΔAUC = +0.164) similarly representing the steepest improvement. Figure 2 illustrates individual dynamic risk trajectories for representative students.

**Table 3:** Landmark-specific predictive performance of the Random Forest Supermodel.

Gender	Landmark	*n*	n_events	event_rate_pct	auc_roc	brier_score
Male	Year 1	2,745	478	17.4	0.751	0.180
Male	Year 2	2,745	478	17.4	0.858	0.135
Male	Year 3	2,745	478	17.4	0.894	0.141
Female	Year 1	1,261	60	4.8	0.616	0.180
Female	Year 2	1,261	60	4.8	0.780	0.093
Female	Year 3	1,261	60	4.8	0.878	0.122

**Figure 2:**
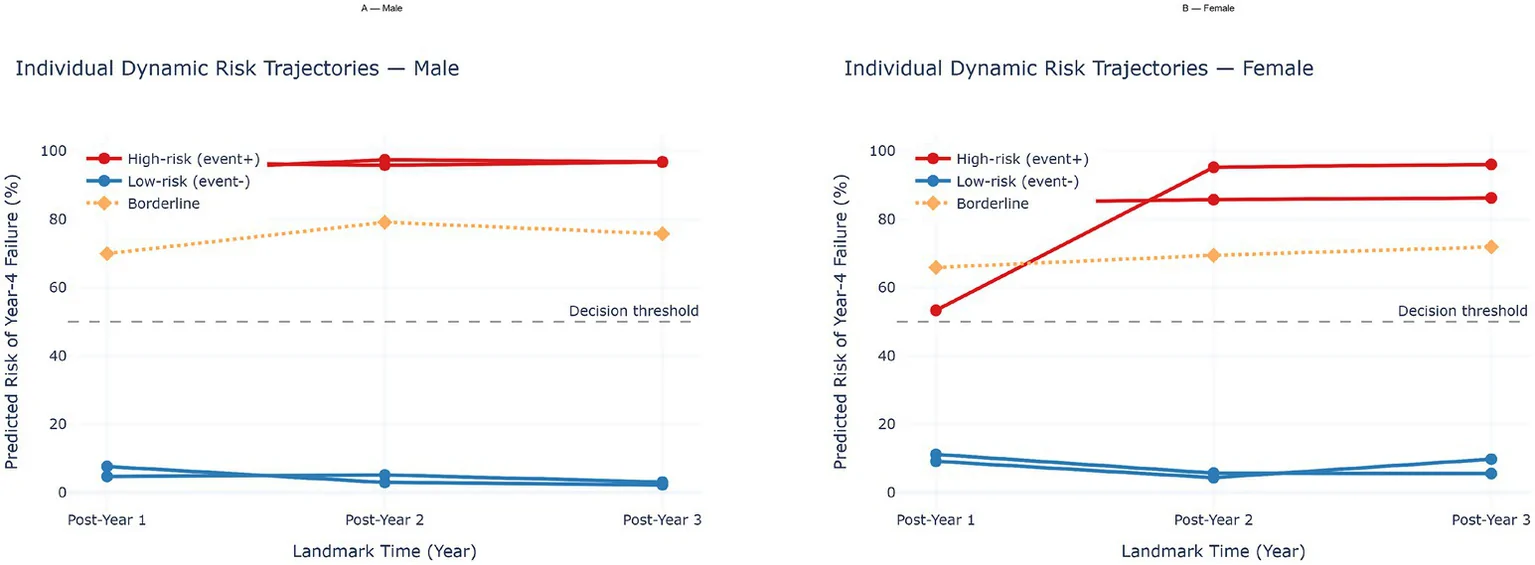
Individual dynamic risk trajectories for representative cases. **(A)** Male, **(B)** Female.

### 3.4 Risk stratification and calibration

Figure 3 presents calibration curves across landmark time points. Overall, the calibration curves demonstrated reasonable agreement between predicted and observed event rates in the medium- and high-risk strata. However, a notable calibration gap was observed in the female Year-3 low-risk stratum, where the observed failure rate was 0.0% (0/421) against a mean predicted risk of 11.3% (Table 4). This upward bias in the lowest-risk female subgroup is consistent with the well-documented challenge of calibrating prediction models in settings with very low outcome incidence (4.8%), where models tend to produce conservative (upwardly biased) risk estimates in the lowest predicted-probability regions. Importantly, the perfect negative predictive value in this stratum (no student classified as low-risk at LM3 experienced failure) indicates that, despite the calibration gap in absolute risk estimates, the model’s risk ranking remains clinically useful for reliably ruling out students who do not require intervention. Numeric calibration assessment via logistic recalibration yielded calibration slopes (ideal = 1) for males of 1.050 (LM1), 1.148 (LM2), and 1.419 (LM3), with corresponding intercepts (ideal = 0) of −1.418, −1.283, and −1.601. For females, calibration slopes were 0.857 (LM1), 1.106 (LM2), and 1.760 (LM3), with intercepts of −2.739, −2.179, and −2.580. Complete calibration statistics for all strata are provided in Supplementary Table S3. Figure 4 displays decision curve analysis demonstrating net benefit across a range of risk thresholds.

**Figure 3:**
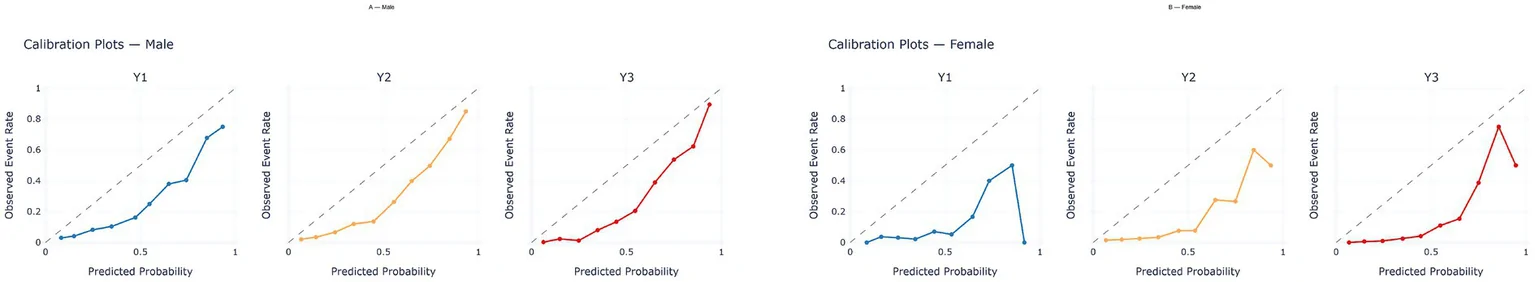
Calibration plots by landmark time point. **(A)** Male, **(B)** Female.

**Table 4:** Risk stratification by predicted probability tertile at LM3 (Random Forest).

Gender	Landmark	risk_group	*n*	n_events	observed_rate_pct	predicted_risk_pct
Male	Year 1	Low	915	52	5.7	18.6
Male	Year 1	Med	942	144	15.3	42.6
Male	Year 1	High	888	282	31.8	61.6
Male	Year 2	Low	915	21	2.3	9.5
Male	Year 2	Med	915	76	8.3	27.4
Male	Year 2	High	915	381	41.6	66.3
Male	Year 3	Low	915	12	1.3	11.3
Male	Year 3	Med	915	61	6.7	33.6
Male	Year 3	High	915	405	44.3	69.1
Female	Year 1	Low	421	14	3.3	22.1
Female	Year 1	Med	462	21	4.5	41.7
Female	Year 1	High	378	25	6.6	55.7
Female	Year 2	Low	421	7	1.7	10
Female	Year 2	Med	420	10	2.4	20.3
Female	Year 2	High	420	43	10.2	44.7
Female	Year 3	Low	421	0	0	11.3
Female	Year 3	Med	420	9	2.1	27.7
Female	Year 3	High	420	51	12.1	53.6

**Figure 4:**
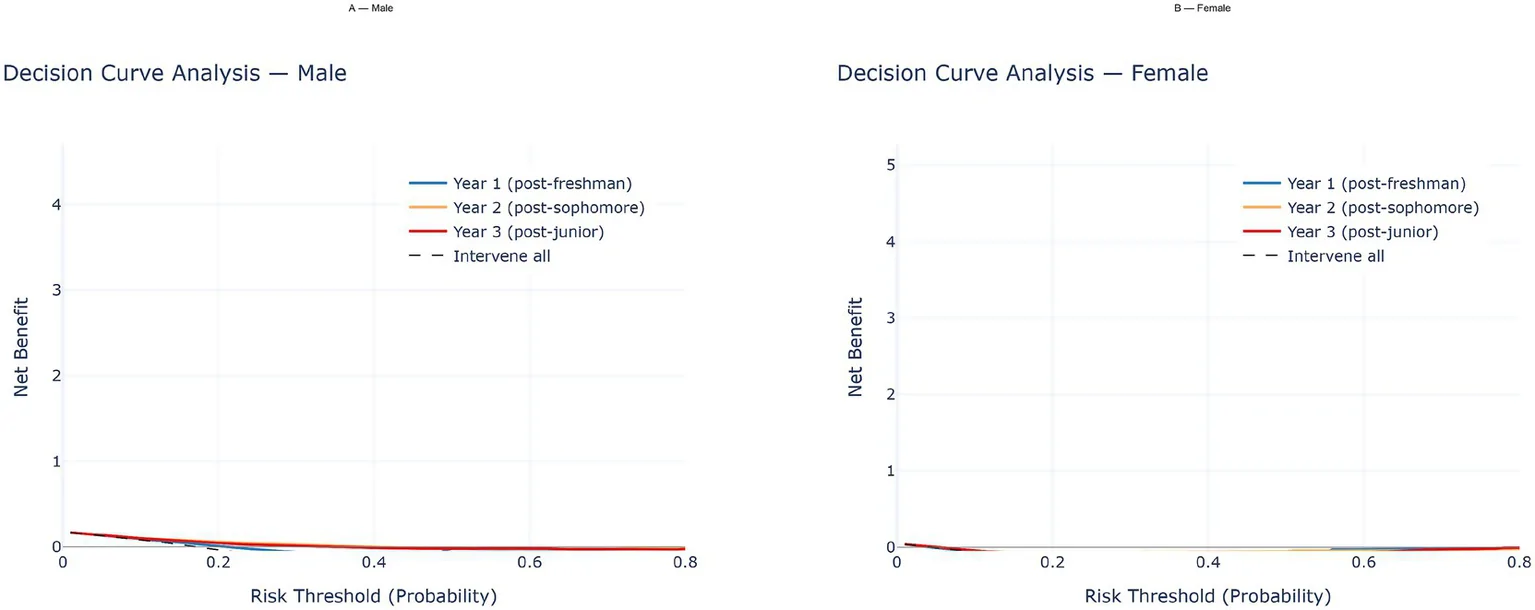
Decision curve analysis by landmark time point. **(A)** Male, **(B)** Female.

### 3.5 Feature importance and predictor patterns

Feature importance analysis revealed that current-year physical fitness indicators carried the greatest predictive weight. For males, the top five predictors at LM3 were: junior-year total score, endurance run time, vital capacity, vital capacity slope, and BMI delta from baseline (Figure 5). Among dynamic features, change-from-baseline in total score and BMI slope were important contributors, supporting the value of longitudinal trajectory information. The vital capacity-to-weight ratio and BMI × vital capacity interaction term provided additional predictive value, suggesting that the combination of elevated body mass and reduced respiratory fitness represents a particularly high-risk phenotype. Figure 6 presents the nomogram offering a simplified point-based risk scoring tool for practical use. The L1-penalized logistic regression model underlying the nomogram achieved a cross-validated AUC of 0.842 (95% CI: 0.831–0.852) for males and 0.725 (95% CI: 0.678–0.767) for females, with Brier scores of 0.159 and 0.195, respectively. By landmark, the logistic model AUC values for males were 0.761 (LM1), 0.856 (LM2), and 0.890 (LM3); corresponding values for females were 0.601 (LM1), 0.731 (LM2), and 0.819 (LM3). Full performance statistics are provided in Supplementary Table S4. The ΔAUC between the Random Forest Supermodel and the logistic nomogram was approximately 0.001 for males and 0.048 for females in the overall comparison, indicating that the nomogram achieves near-equivalent discrimination for males while incurring a modest accuracy penalty for females. This accuracy–accessibility trade-off is discussed further in Section 4.

**Figure 5:**
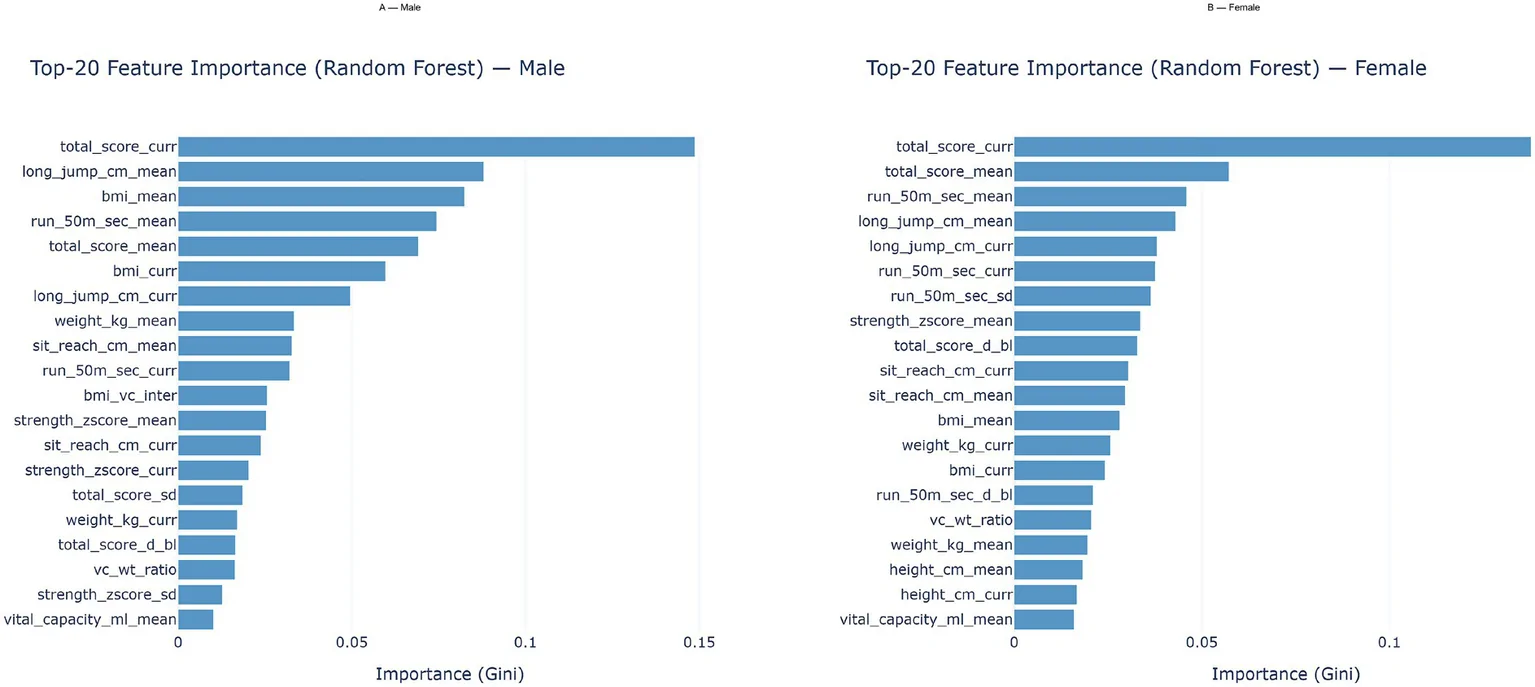
Top-20 feature importance from Random Forest model (Gini importance). **(A)** Male, **(B)** Female.

**Figure 6:**
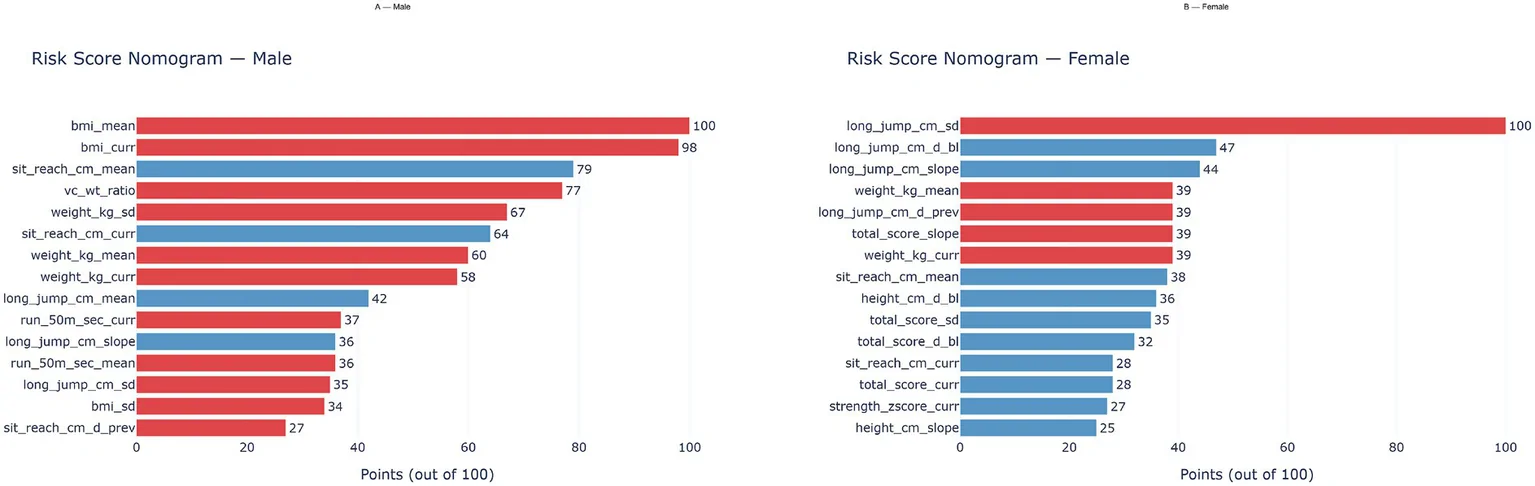
Risk score nomogram based on L1-penalized logistic regression. **(A)** Male, **(B)** Female.

### 3.6 Sensitivity analyses

Sensitivity analyses confirmed the robustness of the primary findings. Complete-case analysis restricted to students with four-year complete data (*n* = 1,786) yielded comparable AUC values (male LM3 AUC = 0.881, female LM3 AUC = 0.863). δ-adjustment analyses under progressively more pessimistic missing-data assumptions (δ = −0.5, −1.0, −1.5 SD) did not substantially alter predictive performance or the identification of the sophomore year as the optimal intervention window.

## 4 Discussion

This four-year longitudinal study developed and validated a Landmark Supermodel for dynamic risk prediction of physical fitness failure in college students. Three main findings emerged. First, the Random Forest Landmark Supermodel achieved strong predictive performance, with AUC values of 0.842 for males and 0.773 for females at the junior-year landmark. Second, prediction accuracy improved substantially with accumulating longitudinal information, with the largest gain occurring between freshman and sophomore years (ΔAUC = +0.107 for males, +0.164 for females), identifying the sophomore year as the optimal intervention window. Third, risk stratification at LM3 effectively separated students into clinically meaningful groups, with a 44.3% failure rate in the male high-risk stratum compared to 1.3% in the low-risk stratum.

These findings extend prior longitudinal research on college student physical fitness in several important ways. Previous studies have documented the continuous rise in BMI across the college period (12, 13), identified distinct physical fitness developmental subtypes through group-based trajectory modeling (29), and examined the mediating role of vital capacity in BMI-fitness associations (30). While those studies characterized population-level trends and retrospective subgroups, they did not address the prospective prediction problem: given a specific student’s data up to a given time point, what is the probability of a future adverse outcome? The Landmark Supermodel fills this gap by providing dynamic, individualized risk estimates that are updated as new data become available.

The identification of the sophomore year as the optimal intervention window has important practical implications. The AUC improvement from 0.751 to 0.858 means that by the end of the second year, the model can identify at-risk students with substantially greater accuracy than at baseline, while still leaving two full academic years for intervention before the senior-year outcome. This aligns with developmental perspectives suggesting that the sophomore year is a period of lifestyle consolidation, when initial college adjustment has stabilized but long-term habits remain malleable (7, 31). From an intervention design perspective, the sophomore year represents a “sweet spot” where predictive certainty and remaining intervention time are both favorable.

The sex-stratified analysis revealed notable differences in both model performance and risk patterns. The lower AUC for females (0.773 vs. 0.842 for males) is primarily attributable to the substantially lower base rate of fitness failure in females (4.8% vs. 17.4%), a well-recognized challenge in imbalanced classification (32). Despite this, the female model achieved a perfect negative predictive value at LM3 for the low-risk stratum, effectively ruling out students who do not require intervention. The smaller BMI increase in females (0.29 vs. 1.08 kg/m^2^ in males) supports the necessity of sex-stratified modeling approaches (33).

From a methodological standpoint, this study introduces the Landmark Supermodel framework to physical fitness assessment. Landmark analysis, widely used in clinical prediction modeling (18, 20, 21), is particularly well-suited to educational settings where data accumulate at fixed annual intervals and decisions must be updated sequentially. The Supermodel variant pools observations across landmark time points, borrows statistical strength, and directly quantifies the incremental value of additional longitudinal data. To our knowledge, this is the first application of landmark methodology to physical fitness prediction in a university setting. The integration of SHAP-based model interpretation (28) and decision curve analysis (26, 27) further enhances the transparency and clinical applicability of the machine learning approach, addressing the “black box” concern that has limited the adoption of predictive models in educational practice (34).

The practical implications extend beyond individual risk prediction. The nomogram presented in Figure 6 provides a transparent, point-based scoring system that physical education teachers can apply without computational resources. The risk stratification table enables targeted resource allocation: students in the high-risk stratum warrant intensive intervention, while those in the low-risk stratum can be safely monitored with routine testing alone. This precision-education approach parallels developments in precision medicine (35), where individualized risk scores guide resource allocation and intervention intensity. In resource-constrained university physical education programs, such tools could enable targeted deployment of supplementary training, nutritional counseling, and lifestyle modification programs to the students who need them most.

This study has several limitations. First, the observational design precludes causal inference; the identified risk factors are predictive markers rather than proven causal determinants. Second, the sample was drawn from a single university in eastern China, which may limit generalizability to other regions and institutional types. Third, key behavioral covariates—dietary intake, extracurricular physical activity, sleep patterns, and screen time—were unavailable and could further improve prediction if incorporated. Fourth, vital capacity is an indirect measure of cardiorespiratory fitness; direct measures such as maximal oxygen uptake (VO₂max) were not available. Fifth, the female model was constrained by the low event rate (4.8%), limiting statistical power. Sixth, external validation in independent cohorts is essential before educational deployment. Seventh, model updating strategies require investigation for sustained performance across successive student cohorts (21). Fourth, the female models were trained on 60 failure events across 57 engineered features, yielding approximately one event per variable (EPV). This falls below the conventional EPV threshold of 10–20 for stable logistic regression model fitting (36), and constitutes a distinct constraint on the female-specific findings. Although the L1 penalty in the logistic nomogram model performs implicit feature selection, effectively reducing the degrees of freedom, and the Random Forest’s ensemble structure provides inherent regularization through bootstrapping and random subspace sampling, the female model estimates should be interpreted with appropriate caution pending external validation in larger female cohorts with higher event rates.

Future research should pursue external validation across multiple universities and regions to establish model transportability. The integration of additional data sources—including wearable device data, campus consumption records, and class attendance logs—could further enhance prediction and move toward a more comprehensive digital twin of student health. Intervention trials embedding the Landmark Supermodel as a risk-based allocation tool would provide the strongest test of its practical utility. Extending the prediction window beyond graduation into early adulthood could connect university fitness assessments with long-term health outcomes, bridging the gap between educational and clinical risk prediction.

## 5 Conclusion

Dynamic landmark risk prediction can accurately identify college students at risk of physical fitness failure by senior year, with AUC reaching 0.894 for males and 0.878 for females at the junior-year landmark. The sophomore year represents the optimal intervention window, where predictive certainty and remaining intervention time are both maximized. The Landmark Supermodel provides an evidence-based, interpretable tool for individualized early warning, enabling physical education teachers to move from group-level surveillance to precision-targeted intervention. This framework demonstrates the feasibility and value of applying clinical prediction methodology to educational fitness assessment, with potential applications in resource allocation, curriculum design, and student health management.

## Data Availability

The original contributions presented in the study are included in the article/Supplementary material, further inquiries can be directed to the corresponding author.
