# Supplementary material for: Dynamic risk prediction of physical fitness failure in college students: a four-year longitudinal landmark supermodel study
Source: Front Public Health. 2026 Sep 23;14:1921844. doi: 10.3389/fpubh.2026.1921844 (PMC13642814; doi:10.3389/fpubh.2026.1921844)
Supplement: Supplementary file 1 [file Supplementary_file_1.DOCX]

**Supplementary Code**

Dynamic Risk Prediction of Physical Fitness Failure in College Students

A Four-Year Longitudinal Landmark Supermodel Study

**FILE: 01_data_cleaning.py**

"""

01_data_cleaning.py

1. 读取 .xls 原始文件（5个Sheet：大一/大二/大三/大四/完整数据）

4. 变量标准化：分性别处理 800m/1000m、引体向上/仰卧起坐

- analysis/data_long.csv

- analysis/data_wide.csv

- analysis/data_analysis.csv

- analysis/data_summary.csv

解析单个学年的Sheet。

表头占2行（Row1=项目名, Row2=成绩/得分），跳过Row2，用Row1的映射。

sheet_names = ['大一', '大二', '大三', '大四']

all_dfs = []

for sheet in sheet_names:

try:

df_sheet = parse_sheet(DATA_PATH, sheet)

all_dfs.append(df_sheet)

except Exception as e:

print(f" [{sheet}] 读取失败: {e}")

df_long = pd.concat(all_dfs, ignore_index=True)

return df_long

def convert_types(df):

numeric_cols = [

'total_score', 'height_cm', 'weight_kg', 'bmi',

'vital_capacity_ml', 'run_50m_sec',

'long_jump_cm', 'sit_reach_cm',

'run_800m_time', 'run_1000m_time',

'situp_count', 'pullup_count'

]

for col in numeric_cols:

if col in df.columns:

df[col] = pd.to_numeric(df[col], errors='coerce')

if 'gender' in df.columns:

df['gender'] = df['gender'].astype(str).str.strip()

df['gender'] = df['gender'].map({'男': 'male', '女': 'female'}).fillna(df['gender'])

if 'grade_level' in df.columns:

df['grade_level'] = df['grade_level'].astype(str).str.strip()

df = df[~df['grade_level'].isin(['等级', 'nan', ''])]

df['student_id'] = df['student_id'].astype(str).str.strip()

year_map = {'大一': 1, '大二': 2, '大三': 3, '大四': 4}

df['year_num'] = df['year_label'].map(year_map)

return df

def clean_anomalies(df):

if 'height_cm' in df.columns:

df.loc[(df['height_cm'] < 130) | (df['height_cm'] > 220), 'height_cm'] = np.nan

if 'weight_kg' in df.columns:

df.loc[(df['weight_kg'] < 30) | (df['weight_kg'] > 200), 'weight_kg'] = np.nan

if 'bmi' in df.columns:

df.loc[(df['bmi'] < 12) | (df['bmi'] > 50), 'bmi'] = np.nan

if 'vital_capacity_ml' in df.columns:

df.loc[(df['vital_capacity_ml'] < 500) | (df['vital_capacity_ml'] > 8000), 'vital_capacity_ml'] = np.nan

if 'run_50m_sec' in df.columns:

df.loc[(df['run_50m_sec'] < 5) | (df['run_50m_sec'] > 20), 'run_50m_sec'] = np.nan

if 'long_jump_cm' in df.columns:

df.loc[(df['long_jump_cm'] < 50) | (df['long_jump_cm'] > 350), 'long_jump_cm'] = np.nan

if 'sit_reach_cm' in df.columns:

df.loc[(df['sit_reach_cm'] < -30) | (df['sit_reach_cm'] > 50), 'sit_reach_cm'] = np.nan

if 'total_score' in df.columns:

df.loc[(df['total_score'] < 0) | (df['total_score'] > 120), 'total_score'] = np.nan

return df

def unify_gender_specific_tests(df):

def time_str_to_sec(val):

if pd.isna(val):

return np.nan

val_str = str(val).strip()

if "'" in val_str:

parts = val_str.replace('"', '').split("'")

try:

minutes = float(parts[0])

seconds = float(parts[1]) if len(parts) > 1 else 0

return minutes * 60 + seconds

except:

return np.nan

else:

try:

return float(val_str)

except:

return np.nan

df['endurance_run_sec'] = np.nan

if 'run_1000m_time' in df.columns:

male_mask = df['gender'] == 'male'

df.loc[male_mask, 'endurance_run_sec'] = df.loc[male_mask, 'run_1000m_time'].apply(time_str_to_sec)

if 'run_800m_time' in df.columns:

female_mask = df['gender'] == 'female'

df.loc[female_mask, 'endurance_run_sec'] = df.loc[female_mask, 'run_800m_time'].apply(time_str_to_sec)

df.loc[(df['endurance_run_sec'] < 120) | (df['endurance_run_sec'] > 600), 'endurance_run_sec'] = np.nan

df['strength_raw'] = np.nan

male_mask = df['gender'] == 'male'

female_mask = df['gender'] == 'female'

if 'pullup_count' in df.columns:

df.loc[male_mask, 'strength_raw'] = df.loc[male_mask, 'pullup_count']

if 'situp_count' in df.columns:

df.loc[female_mask, 'strength_raw'] = df.loc[female_mask, 'situp_count']

for gender in ['male', 'female']:

mask = df['gender'] == gender

if mask.sum() > 0:

mean_val = df.loc[mask, 'strength_raw'].mean()

std_val = df.loc[mask, 'strength_raw'].std()

if std_val > 0:

df.loc[mask, 'strength_zscore'] = (df.loc[mask, 'strength_raw'] - mean_val) / std_val

return df

def define_outcome(df):

senior = df[df['year_num'] == 4][['student_id', 'total_score', 'bmi', 'grade_level']].copy()

senior.columns = ['student_id', 'score_y4', 'bmi_y4', 'grade_y4']

freshman = df[df['year_num'] == 1][['student_id', 'total_score', 'bmi']].copy()

freshman.columns = ['student_id', 'score_y1', 'bmi_y1']

df = df.merge(senior, on='student_id', how='left')

df = df.merge(freshman, on='student_id', how='left')

df['outcome_fail'] = (df['score_y4'] < 60).astype(int)

df['outcome_fail'] = df['outcome_fail'].where(df['score_y4'].notna(), other=np.nan)

df['outcome_bmi_fail'] = ((df['bmi_y4'] >= 28) & (df['score_y4'] < 60)).astype(int)

df['outcome_bmi_fail'] = df['outcome_bmi_fail'].where(df['bmi_y4'].notna() & df['score_y4'].notna(), other=np.nan)

df['score_decline'] = df['score_y1'] - df['score_y4']

df['outcome_worsen'] = ((df['score_y4'] < 60) & (df['score_decline'] >= 10)).astype(int)

df['outcome_worsen'] = df['outcome_worsen'].where(

df['score_y4'].notna() & df['score_y1'].notna(), other=np.nan

)

return df

def filter_valid_students(df, min_years=2, min_measures_per_year=4):

core_measures = ['height_cm', 'weight_kg', 'vital_capacity_ml',

'run_50m_sec', 'long_jump_cm', 'sit_reach_cm', 'endurance_run_sec']

available = [c for c in core_measures if c in df.columns]

df['n_measures'] = df[available].notna().sum(axis=1)

df['valid_year'] = df['n_measures'] >= min_measures_per_year

student_valid_years = df.groupby('student_id')['valid_year'].sum()

valid_students = student_valid_years[student_valid_years >= min_years].index

df_filtered = df[df['student_id'].isin(valid_students)].copy()

print(f" 筛选前: {df['student_id'].nunique()} 人")

print(f" 筛选后 (≥{min_years}年, 每年≥{min_measures_per_year}项): {df_filtered['student_id'].nunique()} 人")

return df_filtered

def build_wide_table(df_long):

pivot_vars = [

'total_score', 'grade_level', 'height_cm', 'weight_kg', 'bmi',

'vital_capacity_ml', 'run_50m_sec', 'long_jump_cm', 'sit_reach_cm',

'endurance_run_sec', 'strength_raw', 'strength_zscore', 'n_measures'

]

available_vars = [v for v in pivot_vars if v in df_long.columns]

static_vars = ['student_id', 'gender', 'college', 'ethnicity_code']

static_available = [v for v in static_vars if v in df_long.columns]

df_static = df_long[static_available].drop_duplicates(subset='student_id')

outcome_vars = ['student_id', 'outcome_fail', 'outcome_bmi_fail', 'outcome_worsen',

'score_y4', 'bmi_y4', 'grade_y4', 'score_y1', 'bmi_y1', 'score_decline']

outcome_available = [v for v in outcome_vars if v in df_long.columns]

df_outcome = df_long[outcome_available].drop_duplicates(subset='student_id')

df_wide = df_static.copy()

for var in available_vars:

temp = df_long.pivot(index='student_id', columns='year_num', values=var)

temp.columns = [f'{var}_y{int(c)}' for c in temp.columns]

df_wide = df_wide.merge(temp, on='student_id', how='left')

df_wide = df_wide.merge(df_outcome, on='student_id', how='left')

return df_wide

def generate_summary(df_long, df_wide):

summary = []

summary.append({'metric': 'total_students_raw', 'value': df_long['student_id'].nunique()})

summary.append({'metric': 'total_records_raw', 'value': len(df_long)})

for yr in [1, 2, 3, 4]:

yr_data = df_long[df_long['year_num'] == yr]

summary.append({'metric': f'n_students_y{yr}', 'value': yr_data['student_id'].nunique()})

for var in ['height_cm', 'weight_kg', 'bmi', 'vital_capacity_ml',

'run_50m_sec', 'endurance_run_sec']:

if var in yr_data.columns:

n_valid = yr_data[var].notna().sum()

summary.append({'metric': f'n_{var}_y{yr}', 'value': n_valid})

for g in df_wide['gender'].unique():

summary.append({'metric': f'n_gender_{g}', 'value': (df_wide['gender'] == g).sum()})

for outcome in ['outcome_fail', 'outcome_bmi_fail', 'outcome_worsen']:

if outcome in df_wide.columns:

n_event = df_wide[outcome].sum()

summary.append({'metric': f'n_{outcome}', 'value': int(n_event)})

df_summary = pd.DataFrame(summary)

return df_summary

def main():

print("=" * 60)

print("东南大学体测数据 — 纵向数据清洗")

print("=" * 60)

print("\n[1/6] 读取原始数据...")

df_long = read_all_sheets()

print(f" 总计: {len(df_long)} 条记录, {df_long['student_id'].nunique()} 名学生")

print("\n[2/6] 数据类型转换与清理...")

df_long = convert_types(df_long)

df_long = clean_anomalies(df_long)

print("\n[3/6] 性别特异性变量统一...")

df_long = unify_gender_specific_tests(df_long)

print("\n[4/6] 定义结局变量...")

df_long = define_outcome(df_long)

print("\n[5/6] 筛选有效学生（≥2年有效测量）...")

df_long = filter_valid_students(df_long, min_years=2, min_measures_per_year=4)

print("\n[6/6] 构建宽表与摘要...")

df_wide = build_wide_table(df_long)

df_summary = generate_summary(df_long, df_wide)

print("\n保存输出文件...")

os.makedirs(OUT_DIR, exist_ok=True)

df_long.to_csv(os.path.join(OUT_DIR, 'data_long.csv'), index=False, encoding='utf-8-sig')

print(f" [OK] data_long.csv ({len(df_long)} rows x {len(df_long.columns)} cols)")

df_wide.to_csv(os.path.join(OUT_DIR, 'data_wide.csv'), index=False, encoding='utf-8-sig')

print(f" [OK] data_wide.csv ({len(df_wide)} rows x {len(df_wide.columns)} cols)")

df_summary.to_csv(os.path.join(OUT_DIR, 'data_summary.csv'), index=False, encoding='utf-8-sig')

print(f" [OK] data_summary.csv")

print("\n" + "=" * 60)

print("数据清洗完成 — 关键指标")

print("=" * 60)

print(f" 纳入学生: {df_wide['student_id'].nunique()}")

print(f" 男性: {(df_wide['gender']=='male').sum()}")

print(f" 女性: {(df_wide['gender']=='female').sum()}")

if 'outcome_fail' in df_wide.columns:

print(f" 大四不及格 (outcome_fail): {int(df_wide['outcome_fail'].sum())} ({df_wide['outcome_fail'].mean()*100:.1f}%)")

if 'outcome_bmi_fail' in df_wide.columns:

print(f" 大四肥胖+不及格 (outcome_bmi_fail): {int(df_wide['outcome_bmi_fail'].sum())} ({df_wide['outcome_bmi_fail'].mean()*100:.1f}%)")

if 'outcome_worsen' in df_wide.columns:

print(f" 大四恶化 (outcome_worsen): {int(df_wide['outcome_worsen'].sum())} ({df_wide['outcome_worsen'].mean()*100:.1f}%)")

return df_long, df_wide, df_summary

if __name__ == '__main__':

df_long, df_wide, df_summary = main()

**FILE: 02_missing_imputation.py**

"""

02_missing_imputation.py

1. Primary: MICE (Multiple Imputation by Chained Equations), m=20, assuming MAR

2. Sensitivity 1: Complete-case analysis (仅保留全部测量者)

3. Sensitivity 2: δ-adjustment pattern-mixture model (应对 MNAR)

关键: 对 outcome 和 student_id 不插补，仅对预测因子插补。

- analysis/data_imputed_male.csv

- analysis/data_imputed_female.csv

- analysis/imputation_diagnostics.csv

- analysis/sensitivity_results.csv

为论文 Table 1 提供数据

使用 BayesianRidge 作为回归器（连续变量）

使用 IterativeImputer = sklearn 的 MICE 实现

对假设 MNAR 的情况做 δ-调整

假设"未测量者体能更差"：对插补的 total_score 减去 δ*SD

delta_values: 从插补值中减去的标准差倍数

- -0.5: 适度悲观调整

- -1.0: 中等悲观调整

- -1.5: 高度悲观调整

**FILE: 03_feature_engineering.py**

"""

03_feature_engineering.py

=

纵向特征工程 + Landmark Supermodel 数据集构建

核心概念 — Landmark Supermodel:

B. 快照特征（landmark 当年的测量值）

C. 累积特征（截至 landmark 的均值/最大值）

E. 波动特征（SD、range）

F. 交互特征（BMI×肺活量体重比等）

- analysis/landmark_data_male.csv

- analysis/landmark_data_female.csv

- analysis/feature_catalog.csv

path = os.path.join(OUT_DIR, 'data_imputed.csv')

if os.path.exists(path):

df = pd.read_csv(path, encoding='utf-8-sig')

print(f"加载插补数据: {len(df)} 行, {df['student_id'].nunique()} 人")

return df

else:

path = os.path.join(OUT_DIR, 'data_long.csv')

df = pd.read_csv(path, encoding='utf-8-sig')

print(f"加载清洗数据 (未插补): {len(df)} 行, {df['student_id'].nunique()} 人")

return df

def add_derived_variables(df):

if 'vital_capacity_ml' in df.columns and 'weight_kg' in df.columns:

df['vital_capacity_ratio'] = df['vital_capacity_ml'] / df['weight_kg'].replace(0, np.nan)

if 'bmi' in df.columns:

df['bmi_category'] = pd.cut(

df['bmi'],

bins=[0, 18.5, 24, 28, 100],

labels=['underweight', 'normal', 'overweight', 'obese']

)

return df

def build_landmark_dataset(df_gender, gender_label):

print(f"\n [{gender_label}] 构建 Landmark 数据集...")

students = df_gender['student_id'].unique()

all_landmarks = []

for sid in students:

stu_data = df_gender[df_gender['student_id'] == sid].sort_values('year_num')

y4_data = stu_data[stu_data['year_num'] == 4]

if len(y4_data) == 0:

continue

outcome_fail = y4_data['total_score'].values[0]

outcome_fail = 1 if (outcome_fail < 60) else 0

static = {

'student_id': sid,

'ethnicity_code': stu_data['ethnicity_code'].iloc[0] if 'ethnicity_code' in stu_data.columns else np.nan,

'college': stu_data['college'].iloc[0] if 'college' in stu_data.columns else 'unknown',

}

for lm_time in [1, 2, 3]:

lm_year_data = stu_data[stu_data['year_num'] <= lm_time].copy()

if len(lm_year_data) == 0:

continue

row = {

'student_id': sid,

'landmark_time': lm_time,

'outcome_fail': outcome_fail,

'n_years_available': len(lm_year_data),

**static,

}

current_year = lm_year_data[lm_year_data['year_num'] == lm_time]

if len(current_year) > 0:

for var in MEASURE_VARS:

if var in current_year.columns:

row[f'{var}_current'] = current_year[var].values[0]

for var in MEASURE_VARS:

if var in lm_year_data.columns:

vals = lm_year_data[var].dropna()

if len(vals) > 0:

row[f'{var}_mean'] = vals.mean()

row[f'{var}_max'] = vals.max()

row[f'{var}_min'] = vals.min()

baseline = lm_year_data[lm_year_data['year_num'] == 1]

if len(baseline) > 0:

for var in MEASURE_VARS:

if var in current_year.columns and var in baseline.columns:

bv = baseline[var].values[0]

cv = current_year[var].values[0] if len(current_year) > 0 else np.nan

if pd.notna(bv) and pd.notna(cv):

row[f'{var}_delta_baseline'] = cv - bv

if lm_time > 1:

prev_year = lm_year_data[lm_year_data['year_num'] == lm_time - 1]

if len(prev_year) > 0 and len(current_year) > 0:

for var in MEASURE_VARS:

if var in current_year.columns and var in prev_year.columns:

pv = prev_year[var].values[0]

cv = current_year[var].values[0]

if pd.notna(pv) and pd.notna(cv):

row[f'{var}_delta_prev'] = cv - pv

for var in MEASURE_VARS:

if var in lm_year_data.columns:

vals = lm_year_data[var].dropna()

if len(vals) >= 2:

row[f'{var}_sd'] = vals.std()

row[f'{var}_range'] = vals.max() - vals.min()

for var in MEASURE_VARS:

if var in lm_year_data.columns:

vals = lm_year_data[['year_num', var]].dropna()

if len(vals) >= 2:

years = vals['year_num'].values

measures = vals[var].values

if years[-1] > years[0]:

row[f'{var}_slope'] = (measures[-1] - measures[0]) / (years[-1] - years[0])

if 'vital_capacity_ml' in current_year.columns and 'weight_kg' in current_year.columns:

vc = current_year['vital_capacity_ml'].values[0]

wt = current_year['weight_kg'].values[0]

if pd.notna(vc) and pd.notna(wt) and wt > 0:

row['vc_weight_ratio'] = vc / wt

if 'bmi_current' in row and row.get('vital_capacity_ml_current'):

if pd.notna(row['bmi_current']) and pd.notna(row['vital_capacity_ml_current']):

row['bmi_x_vc_interaction'] = row['bmi_current'] * (1 / (row['vital_capacity_ml_current'] + 1))

if row.get('bmi_current') and row.get('total_score_delta_baseline'):

if pd.notna(row['bmi_current']) and pd.notna(row['total_score_delta_baseline']):

row['risk_flag_bmi_score'] = 1 if (row['bmi_current'] >= 24 and row['total_score_delta_baseline'] < -5) else 0

all_landmarks.append(row)

df_lm = pd.DataFrame(all_landmarks)

df_lm = df_lm.drop_duplicates(subset=['student_id', 'landmark_time'])

print(f"	生成 {len(df_lm)} 行 (≈ {len(df_lm)//3} 人 × 3 landmarks)")

return df_lm

def clean_feature_matrix(df_lm):

df = df_lm.copy()

id_cols = ['student_id', 'landmark_time', 'outcome_fail', 'n_years_available',

'college', 'ethnicity_code']

feature_cols = [c for c in df.columns if c not in id_cols]

for col in feature_cols:

missing_rate = df[col].isna().mean()

if missing_rate > 0.5:

df = df.drop(columns=[col])

df = df.replace([np.inf, -np.inf], np.nan)

feature_cols_remaining = [c for c in df.columns if c not in id_cols]

for col in feature_cols_remaining:

if df[col].nunique(dropna=True) <= 1:

df = df.drop(columns=[col])

n_features = len([c for c in df.columns if c not in id_cols])

print(f"	最终特征数: {n_features}")

return df

def generate_feature_catalog(df_lm):

id_cols = ['student_id', 'landmark_time', 'outcome_fail', 'n_years_available',

'college', 'ethnicity_code']

feature_cols = [c for c in df_lm.columns if c not in id_cols]

catalog = []

for col in feature_cols:

if col.endswith('_current'):

feat_type = 'Snapshot (current year)'

elif col.endswith('_mean') or col.endswith('_max') or col.endswith('_min'):

feat_type = 'Cumulative summary'

elif col.endswith('_delta_baseline'):

feat_type = 'Change from baseline'

elif col.endswith('_delta_prev'):

feat_type = 'Change from previous'

elif col.endswith('_sd') or col.endswith('_range'):

feat_type = 'Variability'

elif col.endswith('_slope'):

feat_type = 'Linear slope'

elif 'interaction' in col or col.startswith('risk_flag') or col.startswith('vc_weight'):

feat_type = 'Derived / Interaction'

else:

feat_type = 'Other'

n_missing = df_lm[col].isna().sum()

pct_missing = n_missing / len(df_lm) * 100

n_unique = df_lm[col].nunique(dropna=True)

catalog.append({

'feature': col,

'type': feat_type,

'n_missing': n_missing,

'pct_missing': round(pct_missing, 1),

'n_unique': n_unique,

})

df_catalog = pd.DataFrame(catalog)

return df_catalog

def prepare_model_matrix(df_lm):

df = df_lm.copy()

y = df['outcome_fail'].values

id_info = df[['student_id', 'landmark_time']].copy()

drop_cols = ['student_id', 'landmark_time', 'outcome_fail', 'ethnicity_code',

'college', 'n_years_available']

drop_cols = [c for c in drop_cols if c in df.columns]

X = df.drop(columns=drop_cols, errors='ignore')

feature_names = X.columns.tolist()

if 'college' in df.columns:

college_dummies = pd.get_dummies(df['college'], prefix='college', drop_first=True)

X = pd.concat([X, college_dummies], axis=1)

for col in X.columns:

if X[col].isna().any():

X[col] = X[col].fillna(X[col].median())

print(f"	模型矩阵: X={X.shape}, y={len(y)}, 事件率={y.mean()*100:.1f}%")

return X, y, id_info, feature_names

def main():

print("=" * 60)

print("特征工程: Landmark Supermodel 数据集构建")

print("=" * 60)

print("\n[1/5] 加载数据...")

df = load_imputed_data()

print("\n[2/5] 添加衍生变量...")

df = add_derived_variables(df)

if 'outcome_fail' not in df.columns and 'total_score' in df.columns:

senior = df[df['year_num'] == 4][['student_id', 'total_score']].copy()

senior.columns = ['student_id', 'score_y4']

df = df.merge(senior, on='student_id', how='left')

df['outcome_fail'] = (df['score_y4'] < 60).astype(int)

print("\n[3/5] 分性别构建 Landmark 数据集...")

df_male = df[df['gender'] == 'male'].copy()

df_female = df[df['gender'] == 'female'].copy()

df_lm_male = build_landmark_dataset(df_male, 'Male')

df_lm_female = build_landmark_dataset(df_female, 'Female')

print("\n[4/5] 特征清理...")

df_lm_male = clean_feature_matrix(df_lm_male)

df_lm_female = clean_feature_matrix(df_lm_female)

print("\n[5/5] 生成特征目录...")

catalog_male = generate_feature_catalog(df_lm_male)

catalog_female = generate_feature_catalog(df_lm_female)

print("\n准备模型矩阵...")

X_male, y_male, id_male, features_male = prepare_model_matrix(df_lm_male)

X_female, y_female, id_female, features_female = prepare_model_matrix(df_lm_female)

print("\n保存输出文件...")

df_lm_male.to_csv(os.path.join(OUT_DIR, 'landmark_data_male.csv'), index=False, encoding='utf-8-sig')

print(f" [OK] landmark_data_male.csv ({len(df_lm_male)} 行 × {len(df_lm_male.columns)} 列)")

df_lm_female.to_csv(os.path.join(OUT_DIR, 'landmark_data_female.csv'), index=False, encoding='utf-8-sig')

print(f" [OK] landmark_data_female.csv ({len(df_lm_female)} 行 × {len(df_lm_female.columns)} 列)")

catalog_all = pd.concat([catalog_male.assign(gender='male'),

catalog_female.assign(gender='female')])

catalog_all.to_csv(os.path.join(OUT_DIR, 'feature_catalog.csv'), index=False, encoding='utf-8-sig')

print(f" [OK] feature_catalog.csv")

import joblib

joblib.dump({

'X': X_male, 'y': y_male, 'id_info': id_male, 'feature_names': features_male

}, os.path.join(OUT_DIR, 'model_matrix_male.pkl'))

print(f" [OK] model_matrix_male.pkl")

joblib.dump({

'X': X_female, 'y': y_female, 'id_info': id_female, 'feature_names': features_female

}, os.path.join(OUT_DIR, 'model_matrix_female.pkl'))

print(f" [OK] model_matrix_female.pkl")

print("\n" + "=" * 60)

print("特征工程完成 — 关键指标")

print("=" * 60)

print(f" 男性: {df_lm_male['student_id'].nunique()} 人, {len(df_lm_male)} 行, {len(df_lm_male.columns)} 特征")

print(f"	LM1={len(df_lm_male[df_lm_male['landmark_time']==1])} 行")

print(f"	LM2={len(df_lm_male[df_lm_male['landmark_time']==2])} 行")

print(f"	LM3={len(df_lm_male[df_lm_male['landmark_time']==3])} 行")

print(f"	事件率: {y_male.mean()*100:.1f}%")

print(f" 女性: {df_lm_female['student_id'].nunique()} 人, {len(df_lm_female)} 行, {len(df_lm_female.columns)} 特征")

print(f"	事件率: {y_female.mean()*100:.1f}%")

return (X_male, y_male, id_male, features_male,

X_female, y_female, id_female, features_female)

if __name__ == '__main__':

(X_male, y_male, id_male, features_male,

X_female, y_female, id_female, features_female) = main()

**FILE: 04_landmark_modeling.py**

"""

04_landmark_modeling.py

====

Landmark Supermodel — 多模型比较

1. Logistic Regression (L1/L2 elastic-net) — 基准

2. Random Forest — 非线性基准

3. XGBoost — 主力模型

4. (Optional) Gradient Boosting — 补充

Landmark Supermodel 设计:

- 堆叠 LM1+LM2+LM3，landmark_time 作为协变量

- 单一模型可预测任意 landmark 的风险

- 按 student_id 分组的交叉验证（防止同一学生的多个 landmark 行泄露）

- analysis/model_comparison_male.csv

- analysis/model_comparison_female.csv

- analysis/best_model_male.pkl

- analysis/best_model_female.pkl

- analysis/landmark_predictions_male.csv

- analysis/landmark_predictions_female.csv

male_path = os.path.join(OUT_DIR, 'model_matrix_male.pkl')

female_path = os.path.join(OUT_DIR, 'model_matrix_female.pkl')

if os.path.exists(male_path) and os.path.exists(female_path):

data_male = joblib.load(male_path)

data_female = joblib.load(female_path)

print(f"加载模型矩阵: 男 {data_male['X'].shape}, 女 {data_female['X'].shape}")

return data_male, data_female

else:

raise FileNotFoundError("模型矩阵文件未找到。请先运行 03_feature_engineering.py")

def build_models():

models = {

'Logistic_ElasticNet': LogisticRegressionCV(

Cs=10,

cv=5,

penalty='elasticnet',

solver='saga',

l1_ratios=[0.1, 0.3, 0.5, 0.7, 0.9],

max_iter=5000,

random_state=RANDOM_STATE,

class_weight='balanced',

n_jobs=-1

),

'RandomForest': RandomForestClassifier(

n_estimators=500,

max_depth=10,

min_samples_leaf=20,

class_weight='balanced',

random_state=RANDOM_STATE,

n_jobs=-1

),

'XGBoost': xgb.XGBClassifier(

n_estimators=500,

max_depth=5,

learning_rate=0.03,

subsample=0.8,

colsample_bytree=0.8,

scale_pos_weight=1,

eval_metric='logloss',

random_state=RANDOM_STATE,

verbosity=0,

n_jobs=-1

),

}

return models

def evaluate_model(model, X, y, groups, model_name, gender_label):

print(f" [{gender_label}] 评估 {model_name}...")

sgkf = StratifiedGroupKFold(n_splits=N_CV_FOLDS, shuffle=True, random_state=RANDOM_STATE)

if isinstance(model, xgb.XGBClassifier):

neg_weight = (y == 0).sum() / (y == 1).sum()

model.set_params(scale_pos_weight=neg_weight)

y_pred_proba = cross_val_predict(

model, X, y, cv=sgkf, groups=groups,

method='predict_proba', n_jobs=-1

)[:, 1]

y_pred = cross_val_predict(

model, X, y, cv=sgkf, groups=groups,

method='predict', n_jobs=-1

)

auc = roc_auc_score(y, y_pred_proba)

brier = brier_score_loss(y, y_pred_proba)

accuracy = accuracy_score(y, y_pred)

precision = precision_score(y, y_pred, zero_division=0)

recall = recall_score(y, y_pred, zero_division=0)

f1 = f1_score(y, y_pred, zero_division=0)

cm = confusion_matrix(y, y_pred)

tn, fp, fn, tp = cm.ravel()

results = {

'model': model_name,

'gender': gender_label,

'auc_roc': round(auc, 4),

'brier_score': round(brier, 4),

'accuracy': round(accuracy, 4),

'precision': round(precision, 4),

'recall': round(recall, 4),

'f1_score': round(f1, 4),

'specificity': round(tn / (tn + fp), 4) if (tn + fp) > 0 else 0,

'n_samples': len(y),

'n_events': int(y.sum()),

'event_rate': round(y.mean() * 100, 1),

}

return results, y_pred_proba, y_pred

def evaluate_by_landmark(X, y, id_info, y_pred_proba, y_pred, gender_label):

lm_results = []

predictions = id_info.copy()

predictions['y_true'] = y

predictions['y_pred_proba'] = y_pred_proba

predictions['y_pred'] = y_pred

for lm in [1, 2, 3]:

mask = id_info['landmark_time'] == lm

y_lm = y[mask.values]

proba_lm = y_pred_proba[mask.values]

pred_lm = y_pred[mask.values]

if len(y_lm) == 0:

continue

auc = roc_auc_score(y_lm, proba_lm) if y_lm.nunique() > 1 else np.nan

brier = brier_score_loss(y_lm, proba_lm)

acc = accuracy_score(y_lm, pred_lm)

lm_results.append({

'gender': gender_label,

'landmark': lm,

'landmark_label': ['大一后', '大二后', '大三后'][lm-1],

'n': len(y_lm),

'n_events': int(y_lm.sum()),

'auc_roc': round(auc, 4) if not np.isnan(auc) else 'N/A',

'brier_score': round(brier, 4),

'accuracy': round(acc, 4),

})

df_lm = pd.DataFrame(lm_results)

return df_lm, predictions

def train_final_model(model_class, X, y, model_name):

print(f" 训练最终 {model_name} 模型...")

model = model_class

if isinstance(model, xgb.XGBClassifier):

neg_weight = (y == 0).sum() / (y == 1).sum()

model.set_params(scale_pos_weight=neg_weight)

model.fit(X, y)

return model

def extract_feature_importance(model, feature_names, model_name, gender_label):

importances = None

if isinstance(model, LogisticRegressionCV):

importances = np.abs(model.coef_[0]) if len(model.coef_.shape) == 2 else np.abs(model.coef_)

elif isinstance(model, RandomForestClassifier):

importances = model.feature_importances_

elif isinstance(model, xgb.XGBClassifier):

importances = model.feature_importances_

if importances is not None:

df_imp = pd.DataFrame({

'feature': feature_names[:len(importances)],

'importance': importances,

'model': model_name,

'gender': gender_label,

}).sort_values('importance', ascending=False)

return df_imp

return None

def bootstrap_auc_ci(y_true, y_pred_proba, n_bootstrap=1000, alpha=0.05):

rng = np.random.RandomState(RANDOM_STATE)

aucs = []

n = len(y_true)

for _ in range(n_bootstrap):

idx = rng.randint(0, n, n)

y_t = y_true[idx]

y_p = y_pred_proba[idx]

if y_t.nunique() > 1:

aucs.append(roc_auc_score(y_t, y_p))

ci_lower = np.percentile(aucs, alpha/2 * 100)

ci_upper = np.percentile(aucs, (1 - alpha/2) * 100)

return round(ci_lower, 4), round(ci_upper, 4)

def main():

print("=" * 60)

print("Landmark Supermodel — 多模型比较")

print("=" * 60)

print("\n[1/6] 加载模型矩阵...")

data_male, data_female = load_model_matrices()

models = build_models()

all_results = []

all_predictions = {}

all_importances = []

for gender, data in [('Male', data_male), ('Female', data_female)]:

X = data['X']

y = data['y']

id_info = data['id_info']

feature_names = data['feature_names']

if 'landmark_time' in X.columns:

X = X.drop(columns=['landmark_time'])

groups = id_info['student_id'].values

print(f"\n{'='*40}")

print(f" {gender}: N={len(y)}, 事件={int(y.sum())} ({y.mean()*100:.1f}%)")

print(f" 特征数: {len(X.columns)}")

print(f"{'='*40}")

best_model_name = None

best_auc = 0

best_model_obj = None

best_y_proba = None

best_y_pred = None

for model_name, model in models.items():

results, y_pred_proba, y_pred = evaluate_model(

model, X, y, groups, model_name, gender

)

all_results.append(results)

ci_low, ci_high = bootstrap_auc_ci(y, y_pred_proba)

results['auc_ci_low'] = ci_low

results['auc_ci_high'] = ci_high

print(f"	{model_name}: AUC={results['auc_roc']} [95%CI: {ci_low}-{ci_high}], "

f"Brier={results['brier_score']}, F1={results['f1_score']}")

if results['auc_roc'] > best_auc:

best_auc = results['auc_roc']

best_model_name = model_name

best_y_proba = y_pred_proba

best_y_pred = y_pred

best_model_obj = train_final_model(model, X, y, model_name)

print(f"\n 最佳模型: {best_model_name} (AUC={best_auc:.4f})")

df_lm_results, predictions = evaluate_by_landmark(

X, y, id_info, best_y_proba, best_y_pred, gender

)

print(df_lm_results.to_string(index=False))

all_predictions[gender] = predictions

if best_model_obj is not None:

df_imp = extract_feature_importance(

best_model_obj, feature_names, best_model_name, gender

)

if df_imp is not None:

all_importances.append(df_imp)

joblib.dump(

{'model': best_model_obj, 'model_name': best_model_name,

'feature_names': feature_names, 'gender': gender},

os.path.join(OUT_DIR, f'best_model_{gender.lower()}.pkl')

)

print(f" [OK] best_model_{gender.lower()}.pkl")

print("\n[6/6] 保存结果...")

df_all = pd.DataFrame(all_results)

df_all.to_csv(os.path.join(OUT_DIR, 'model_comparison.csv'), index=False, encoding='utf-8-sig')

print(f" [OK] model_comparison.csv")

for gender, preds in all_predictions.items():

preds.to_csv(

os.path.join(OUT_DIR, f'landmark_predictions_{gender.lower()}.csv'),

index=False, encoding='utf-8-sig'

)

print(f" [OK] landmark_predictions_{gender.lower()}.csv")

if all_importances:

df_imp_all = pd.concat(all_importances, ignore_index=True)

df_imp_all.to_csv(os.path.join(OUT_DIR, 'feature_importance.csv'), index=False, encoding='utf-8-sig')

print(f" [OK] feature_importance.csv")

print("\n" + "=" * 60)

print("建模完成 — 模型比较总表")

print("=" * 60)

cols_show = ['model', 'gender', 'auc_roc', 'auc_ci_low', 'auc_ci_high',

'brier_score', 'f1_score', 'recall']

print(df_all[cols_show].to_string(index=False))

return df_all, all_predictions, all_importances

if __name__ == '__main__':

df_all, all_predictions, all_importances = main()

**FILE: 05_evaluation_visualization.py**

"""

05_evaluation_visualization.py

=

产出 (按论文 Figure 顺序):

Fig 1: 研究设计流程图 (conceptual, 手动排版)

Fig 2: ROC 曲线比较 (各模型在同一坐标轴)

Fig 3: Calibration plots (校准曲线)

Fig 4: Decision Curve Analysis (临床净获益)

Fig 5: SHAP 特征重要性 (beeswarm + bar)

Fig 6: 个体化动态风险轨迹 (3 students × 3 landmarks)

Fig 7: Nomogram (基于 Logistic 模型的列线图)

Table 2: 模型比较表 (AUC / Brier / NRI / IDI)

Table 3: 按 landmark 分层的性能

- analysis/figures/*.png (.svg)

- analysis/tables/*.csv

df_comp = pd.read_csv(os.path.join(OUT_DIR, 'model_comparison.csv'), encoding='utf-8-sig')

predictions = {}

for gender in ['male', 'female']:

path = os.path.join(OUT_DIR, f'landmark_predictions_{gender}.csv')

if os.path.exists(path):

predictions[gender] = pd.read_csv(path, encoding='utf-8-sig')

feature_imp_path = os.path.join(OUT_DIR, 'feature_importance.csv')

df_imp = None

if os.path.exists(feature_imp_path):

df_imp = pd.read_csv(feature_imp_path, encoding='utf-8-sig')

return df_comp, predictions, df_imp

def plot_roc_curves(predictions, gender='male'):

preds = predictions.get(gender)

if preds is None:

return

fig, ax = plt.subplots(1, 1, figsize=(5, 4.5))

colors = {1: '#2c7bb6', 2: '#fdae61', 3: '#d7191c'}

labels = {1: 'Year 1 (post-freshman)', 2: 'Year 2 (post-sophomore)', 3: 'Year 3 (post-junior)'}

for lm in [1, 2, 3]:

mask = preds['landmark_time'] == lm

if mask.sum() == 0:

continue

y_true = preds.loc[mask, 'y_true'].values

y_proba = preds.loc[mask, 'y_pred_proba'].values

if len(np.unique(y_true)) < 2:

continue

fpr, tpr, _ = roc_curve(y_true, y_proba)

auc_val = auc(fpr, tpr)

ax.plot(fpr, tpr, color=colors[lm], lw=1.5,

label=f'{labels[lm]} (AUC={auc_val:.3f})', alpha=0.9)

ax.plot([0, 1], [0, 1], 'k--', lw=0.8, alpha=0.4)

ax.set_xlabel('1 - Specificity (False Positive Rate)')

ax.set_ylabel('Sensitivity (True Positive Rate)')

ax.set_title(f'ROC Curves by Landmark Time — {gender.capitalize()}')

ax.legend(loc='lower right', frameon=False)

ax.set_xlim([-0.02, 1.02])

ax.set_ylim([-0.02, 1.02])

plt.tight_layout()

fig.savefig(os.path.join(FIG_DIR, f'fig2_roc_{gender}.png'))

fig.savefig(os.path.join(FIG_DIR, f'fig2_roc_{gender}.svg'))

plt.close()

print(f" [OK] fig2_roc_{gender}.png/svg")

def plot_calibration(predictions, gender='male', n_bins=10):

preds = predictions.get(gender)

if preds is None:

return

fig, axes = plt.subplots(1, 3, figsize=(12, 4))

colors = {1: '#2c7bb6', 2: '#fdae61', 3: '#d7191c'}

labels = {1: 'Year 1', 2: 'Year 2', 3: 'Year 3'}

for idx, lm in enumerate([1, 2, 3]):

ax = axes[idx]

mask = preds['landmark_time'] == lm

y_true = preds.loc[mask, 'y_true'].values

y_proba = preds.loc[mask, 'y_pred_proba'].values

if len(y_true) < 10 or len(np.unique(y_true)) < 2:

ax.text(0.5, 0.5, 'Insufficient data', ha='center', va='center')

continue

prob_true, prob_pred = calibration_curve(y_true, y_proba, n_bins=n_bins)

brier = brier_score_loss(y_true, y_proba)

ax.plot(prob_pred, prob_true, 'o-', color=colors[lm], markersize=4, lw=1.5)

ax.plot([0, 1], [0, 1], 'k--', lw=0.8, alpha=0.4)

ax.set_xlabel('Predicted Probability')

ax.set_ylabel('Observed Event Rate')

ax.set_title(f'{labels[lm]} (Brier={brier:.3f})')

ax.set_xlim([-0.02, 1.02])

ax.set_ylim([-0.02, 1.02])

fig.suptitle(f'Calibration Plots — {gender.capitalize()}', fontsize=11, y=1.01)

plt.tight_layout()

fig.savefig(os.path.join(FIG_DIR, f'fig3_calibration_{gender}.png'))

fig.savefig(os.path.join(FIG_DIR, f'fig3_calibration_{gender}.svg'))

plt.close()

print(f" [OK] fig3_calibration_{gender}.png/svg")

def decision_curve_analysis(y_true, y_pred_proba, thresholds=None):

if thresholds is None:

thresholds = np.linspace(0.01, 0.99, 99)

n = len(y_true)

net_benefit = []

net_benefit_all = []

net_benefit_none = []

for t in thresholds:

y_pred_binary = (y_pred_proba >= t).astype(int)

tp = ((y_pred_binary == 1) & (y_true == 1)).sum()

fp = ((y_pred_binary == 1) & (y_true == 0)).sum()

nb = tp / n - (fp / n) * (t / (1 - t))

nb_all = y_true.mean() - (1 - y_true.mean()) * (t / (1 - t))

nb_none = 0

net_benefit.append(nb)

net_benefit_all.append(nb_all)

net_benefit_none.append(nb_none)

return thresholds, net_benefit, net_benefit_all, net_benefit_none

def plot_decision_curve(predictions, gender='male'):

preds = predictions.get(gender)

if preds is None:

return

fig, ax = plt.subplots(1, 1, figsize=(5.5, 4.5))

colors = {1: '#2c7bb6', 2: '#fdae61', 3: '#d7191c'}

labels = {1: 'Year 1', 2: 'Year 2', 3: 'Year 3'}

for lm in [1, 2, 3]:

mask = preds['landmark_time'] == lm

y_true = preds.loc[mask, 'y_true'].values

y_proba = preds.loc[mask, 'y_pred_proba'].values

if len(y_true) < 10 or len(np.unique(y_true)) < 2:

continue

thresholds, nb, nb_all, nb_none = decision_curve_analysis(y_true, y_proba)

ax.plot(thresholds, nb, color=colors[lm], lw=1.5, label=labels[lm])

thresholds, nb_all, _, _ = decision_curve_analysis(

preds['y_true'].values, preds['y_pred_proba'].values

)

ax.plot(thresholds, nb_all, 'k--', lw=0.8, alpha=0.5, label='Intervene all')

ax.axhline(y=0, color='gray', lw=0.5, alpha=0.3, linestyle='-')

ax.set_xlabel('Risk Threshold (Probability)')

ax.set_ylabel('Net Benefit')

ax.set_title(f'Decision Curve Analysis — {gender.capitalize()}')

ax.legend(loc='best', frameon=False)

ax.set_xlim([0, 0.8])

ax.set_ylim([-0.05, None])

plt.tight_layout()

fig.savefig(os.path.join(FIG_DIR, f'fig4_dca_{gender}.png'))

fig.savefig(os.path.join(FIG_DIR, f'fig4_dca_{gender}.svg'))

plt.close()

print(f" [OK] fig4_dca_{gender}.png/svg")

def plot_shap_analysis(gender='male'):

try:

import shap

except ImportError:

print(" [跳过] SHAP 未安装，请 pip install shap")

return

model_path = os.path.join(OUT_DIR, f'best_model_{gender}.pkl')

data_path = os.path.join(OUT_DIR, f'model_matrix_{gender}.pkl')

if not os.path.exists(model_path) or not os.path.exists(data_path):

print(f" [{gender}] 模型或数据文件缺失，跳过 SHAP")

return

model_data = joblib.load(model_path)

matrix_data = joblib.load(data_path)

model = model_data['model']

X = matrix_data['X']

feature_names = matrix_data['feature_names']

if hasattr(X, 'columns'):

feature_names = X.columns.tolist()

else:

X = pd.DataFrame(X, columns=feature_names)

if 'landmark_time' in X.columns:

X = X.drop(columns=['landmark_time'])

feature_names = [f for f in feature_names if f != 'landmark_time']

print(f" [{gender}] 计算 SHAP 值 (n={len(X)}, p={len(feature_names)})...")

if hasattr(model, 'get_booster'):

explainer = shap.TreeExplainer(model)

shap_values = explainer.shap_values(X[:1000])

elif hasattr(model, 'feature_importances_'):

explainer = shap.TreeExplainer(model)

shap_values = explainer.shap_values(X[:1000])

if isinstance(shap_values, list):

shap_values = shap_values[1]

else:

print("	使用 KernelExplainer (较慢)...")

background = shap.kmeans(X, 50)

explainer = shap.KernelExplainer(model.predict_proba, background)

shap_values = explainer.shap_values(X[:200])[1]

fig, ax = plt.subplots(1, 1, figsize=(7, 6))

shap.summary_plot(

shap_values, X[:1000], feature_names=feature_names,

max_display=20, show=False

)

ax.set_title(f'SHAP Feature Importance — {gender.capitalize()}', fontsize=10)

plt.tight_layout()

fig.savefig(os.path.join(FIG_DIR, f'fig5_shap_beeswarm_{gender}.png'), dpi=300)

fig.savefig(os.path.join(FIG_DIR, f'fig5_shap_beeswarm_{gender}.svg'))

plt.close()

print(f" [OK] fig5_shap_beeswarm_{gender}.png/svg")

fig, ax = plt.subplots(1, 1, figsize=(6, 5))

shap.summary_plot(

shap_values, X[:1000], feature_names=feature_names,

plot_type='bar', max_display=15, show=False

)

ax.set_title(f'SHAP Mean Importance — {gender.capitalize()}', fontsize=10)

plt.tight_layout()

fig.savefig(os.path.join(FIG_DIR, f'fig5_shap_bar_{gender}.png'), dpi=300)

fig.savefig(os.path.join(FIG_DIR, f'fig5_shap_bar_{gender}.svg'))

plt.close()

print(f" [OK] fig5_shap_bar_{gender}.png/svg")

def plot_individual_trajectories(predictions, gender='male', n_cases=3):

preds = predictions.get(gender)

if preds is None:

return

high_risk = preds[preds['y_true'] == 1].groupby('student_id')['y_pred_proba'].max()

low_risk = preds[preds['y_true'] == 0].groupby('student_id')['y_pred_proba'].min()

cases = []

high_ids = high_risk.nlargest(min(5, len(high_risk))).index

cases.append(('High-risk (event+)', high_ids[0]))

mid_risk = preds[(preds['y_true'] == 1)].groupby('student_id')['y_pred_proba'].mean()

if len(mid_risk) > 1:

mid_id = mid_risk.nlargest(len(mid_risk)//2).index[-1]

cases.append(('Medium-risk (event+)', mid_id))

low_ids = low_risk.nsmallest(min(5, len(low_risk))).index

cases.append(('Low-risk (event-)', low_ids[0]))

fig, ax = plt.subplots(1, 1, figsize=(6, 4.5))

colors = {'High': '#d7191c', 'Medium': '#fdae61', 'Low': '#2c7bb6'}

for label, sid in cases:

stu_data = preds[preds['student_id'] == sid].sort_values('landmark_time')

risk_type = label.split(' ')[0]

ax.plot(

stu_data['landmark_time'].values,

stu_data['y_pred_proba'].values * 100,

'o-', lw=2, markersize=7, color=colors.get(risk_type, '#999'),

label=label

)

final_outcome = 'FAIL' if stu_data['y_true'].values[0] == 1 else 'PASS'

ax.annotate(

final_outcome,

xy=(stu_data['landmark_time'].values[-1], stu_data['y_pred_proba'].values[-1] * 100),

xytext=(5, 10), textcoords='offset points',

fontsize=8, fontweight='bold',

color='red' if final_outcome == 'FAIL' else 'green'

)

ax.axhline(y=50, color='gray', linestyle='--', lw=0.8, alpha=0.5, label='Decision threshold (50%)')

ax.set_xlabel('Landmark Time (Year)')

ax.set_ylabel('Predicted Risk of Failure at Year 4 (%)')

ax.set_title(f'Individual Dynamic Risk Trajectories — {gender.capitalize()}')

ax.set_xticks([1, 2, 3])

ax.set_xticklabels(['Post-Year 1', 'Post-Year 2', 'Post-Year 3'])

ax.legend(loc='best', frameon=False)

ax.set_ylim([-5, 105])

plt.tight_layout()

fig.savefig(os.path.join(FIG_DIR, f'fig6_trajectories_{gender}.png'))

fig.savefig(os.path.join(FIG_DIR, f'fig6_trajectories_{gender}.svg'))

plt.close()

print(f" [OK] fig6_trajectories_{gender}.png/svg")

def build_nomogram(gender='male'):

from sklearn.linear_model import LogisticRegression

data_path = os.path.join(OUT_DIR, f'model_matrix_{gender}.pkl')

if not os.path.exists(data_path):

return

matrix_data = joblib.load(data_path)

X = matrix_data['X']

y = matrix_data['y']

feature_names = matrix_data['feature_names']

if hasattr(X, 'columns'):

feature_names = X.columns.tolist()

else:

X = pd.DataFrame(X, columns=feature_names)

lr = LogisticRegression(penalty='l1', solver='saga', C=0.1,

max_iter=5000, random_state=RANDOM_STATE,

class_weight='balanced')

lr.fit(X, y)

coef = lr.coef_[0]

intercept = lr.intercept_[0]

df_nomogram = pd.DataFrame({

'variable': feature_names[:len(coef)],

'coefficient': coef,

'abs_coef': np.abs(coef),

}).sort_values('abs_coef', ascending=False)

df_nomogram = df_nomogram.head(15)

max_abs = df_nomogram['abs_coef'].max()

df_nomogram['points'] = (df_nomogram['abs_coef'] / max_abs * 100).round(0).astype(int)

df_nomogram['direction'] = df_nomogram['coefficient'].apply(

lambda x: 'Risk ↑' if x > 0 else 'Risk ↓'

)

df_nomogram = df_nomogram.head(10)

fig, ax = plt.subplots(1, 1, figsize=(8, 4))

y_pos = range(len(df_nomogram))

bars = ax.barh(y_pos, df_nomogram['points'].values,

color=['#d7191c' if c > 0 else '#2c7bb6' for c in df_nomogram['coefficient']],

alpha=0.8, height=0.6)

ax.set_yticks(y_pos)

ax.set_yticklabels(df_nomogram['variable'].values, fontsize=7)

ax.set_xlabel('Points (out of 100)')

ax.set_title(f'Risk Score Nomogram — {gender.capitalize()}\n'

f'(Total Points = Σ individual scores. Higher total = higher risk)',

fontsize=10)

ax.invert_yaxis()

for i, (pts, direction) in enumerate(zip(df_nomogram['points'], df_nomogram['direction'])):

ax.text(pts + 0.5, i, direction, va='center', fontsize=7, color='gray')

plt.tight_layout()

fig.savefig(os.path.join(FIG_DIR, f'fig7_nomogram_{gender}.png'))

fig.savefig(os.path.join(FIG_DIR, f'fig7_nomogram_{gender}.svg'))

plt.close()

print(f" [OK] fig7_nomogram_{gender}.png/svg")

df_nomogram.to_csv(os.path.join(TABLE_DIR, f'nomogram_{gender}.csv'),

index=False, encoding='utf-8-sig')

return df_nomogram

def build_model_comparison_table(df_comp):

table_cols = ['model', 'gender', 'auc_roc', 'auc_ci_low', 'auc_ci_high',

'brier_score', 'precision', 'recall', 'f1_score', 'specificity']

available = [c for c in table_cols if c in df_comp.columns]

df_table = df_comp[available].copy()

if all(c in df_table.columns for c in ['auc_roc', 'auc_ci_low', 'auc_ci_high']):

df_table['AUC (95% CI)'] = df_table.apply(

lambda r: f"{r['auc_roc']:.3f} ({r['auc_ci_low']:.3f}-{r['auc_ci_high']:.3f})", axis=1

)

df_table = df_table.drop(columns=['auc_roc', 'auc_ci_low', 'auc_ci_high'])

df_table.to_csv(os.path.join(TABLE_DIR, 'table2_model_comparison.csv'),

index=False, encoding='utf-8-sig')

print(f" [OK] table2_model_comparison.csv")

return df_table

def build_landmark_performance_table(predictions):

rows = []

for gender, preds in predictions.items():

for lm in [1, 2, 3]:

mask = preds['landmark_time'] == lm

y_true = preds.loc[mask, 'y_true'].values

y_proba = preds.loc[mask, 'y_pred_proba'].values

if len(y_true) == 0 or len(np.unique(y_true)) < 2:

continue

auc_val = roc_auc_score(y_true, y_proba)

brier = brier_score_loss(y_true, y_proba)

rows.append({

'gender': gender.capitalize(),

'landmark': f'Year {lm}',

'n': len(y_true),

'n_events': int(y_true.sum()),

'event_rate_pct': round(y_true.mean() * 100, 1),

'auc_roc': round(auc_val, 3),

'brier_score': round(brier, 3),

})

df_table = pd.DataFrame(rows)

df_table.to_csv(os.path.join(TABLE_DIR, 'table3_landmark_performance.csv'),

index=False, encoding='utf-8-sig')

print(f" [OK] table3_landmark_performance.csv")

return df_table

def build_risk_stratification_table(predictions, threshold=0.5):

rows = []

for gender, preds in predictions.items():

for lm in [1, 2, 3]:

mask = preds['landmark_time'] == lm

sub = preds[mask].copy()

if len(sub) < 30:

continue

sub['risk_group'] = pd.qcut(sub['y_pred_proba'], q=3,

labels=['Low', 'Medium', 'High'])

for group in ['Low', 'Medium', 'High']:

grp = sub[sub['risk_group'] == group]

if len(grp) > 0:

rows.append({

'gender': gender.capitalize(),

'landmark': f'Year {lm}',

'risk_group': group,

'n': len(grp),

'n_events': int(grp['y_true'].sum()),

'observed_rate_pct': round(grp['y_true'].mean() * 100, 1),

'mean_predicted_risk': round(grp['y_pred_proba'].mean() * 100, 1),

})

df_strat = pd.DataFrame(rows)

df_strat.to_csv(os.path.join(TABLE_DIR, 'risk_stratification.csv'),

index=False, encoding='utf-8-sig')

print(f" [OK] risk_stratification.csv")

return df_strat

def main():

print("=" * 60)

print("模型评估 + SCI 论文可视化")

print("=" * 60)

print("\n[1/9] 加载结果...")

df_comp, predictions, df_imp = load_results()

for gender in ['male', 'female']:

if gender not in predictions or predictions[gender] is None:

print(f" [{gender}] 无预测数据，跳过")

continue

print(f"\n{'='*40}")

print(f" {gender.upper()}")

print(f"{'='*40}")

print("\n[2/9] ROC 曲线...")

plot_roc_curves(predictions, gender)

print("\n[3/9] 校准曲线...")

plot_calibration(predictions, gender)

print("\n[4/9] 决策曲线分析...")

plot_decision_curve(predictions, gender)

print("\n[5/9] SHAP 分析...")

plot_shap_analysis(gender)

print("\n[6/9] 个体动态风险轨迹...")

plot_individual_trajectories(predictions, gender)

print("\n[7/9] Nomogram...")

build_nomogram(gender)

print("\n[8/9] 模型比较表...")

build_model_comparison_table(df_comp)

print("\n[9/9] Landmark 分层性能 + 风险分层...")

build_landmark_performance_table(predictions)

build_risk_stratification_table(predictions)

print("\n" + "=" * 60)

print("所有输出完成")

print(f" 图片: {FIG_DIR}/")

print(f" 表格: {TABLE_DIR}/")

print("=" * 60)

figs = os.listdir(FIG_DIR) if os.path.exists(FIG_DIR) else []

tables = os.listdir(TABLE_DIR) if os.path.exists(TABLE_DIR) else []

print(f" 生成 {len(figs)} 张图, {len(tables)} 个表格")

for f in figs:

print(f"	[FIG] {f}")

for t in tables:

print(f"	[TBL] {t}")

if __name__ == '__main__':

main()

**FILE: 06_revision_analyses.py**

import pandas as pd

import numpy as np

from sklearn.model_selection import StratifiedGroupKFold, cross_val_predict

from sklearn.linear_model import LogisticRegression

from sklearn.ensemble import RandomForestClassifier

from sklearn.metrics import (roc_auc_score, brier_score_loss,

accuracy_score, precision_score, recall_score,

f1_score)

from sklearn.calibration import calibration_curve

import xgboost as xgb

import joblib

import os

import warnings

warnings.filterwarnings('ignore')

OUT_DIR = r"D:\东南大学体测数据\analysis"

TABLE_DIR = os.path.join(OUT_DIR, 'tables')

RANDOM_STATE = 42

N_CV_FOLDS = 10

N_BOOTSTRAP = 500

os.makedirs(TABLE_DIR, exist_ok=True)

def delong_roc_variance(y_true, y_pred_proba):

n = len(y_true)

n_pos = int(y_true.sum())

n_neg = n - n_pos

if n_pos == 0 or n_neg == 0:

return None, None, None

order = np.argsort(-y_pred_proba)

y_sorted = y_true[order]

proba_sorted = y_pred_proba[order]

pos_idx = np.where(y_sorted == 1)[0]

neg_idx = np.where(y_sorted == 0)[0]

V_pos = np.zeros(n_pos)

V_neg = np.zeros(n_neg)

for i, pi in enumerate(pos_idx):

n_less = np.sum(y_pred_proba[y_true == 0] < proba_sorted[pi])

n_equal = np.sum(y_pred_proba[y_true == 0] == proba_sorted[pi])

V_pos[i] = (n_less + 0.5 * n_equal) / n_neg

for i, ni in enumerate(neg_idx):

n_greater = np.sum(y_pred_proba[y_true == 1] > proba_sorted[ni])

n_equal = np.sum(y_pred_proba[y_true == 1] == proba_sorted[ni])

V_neg[i] = (n_greater + 0.5 * n_equal) / n_pos

return V_pos, V_neg, n_pos, n_neg

def delong_test(y_true, proba_a, proba_b):

auc_a = roc_auc_score(y_true, proba_a)

auc_b = roc_auc_score(y_true, proba_b)

n = len(y_true)

n_pos = int(y_true.sum())

n_neg = n - n_pos

V_pos_a, V_neg_a, _, _ = delong_roc_variance(y_true, proba_a)

V_pos_b, V_neg_b, _, _ = delong_roc_variance(y_true, proba_b)

if V_pos_a is None or V_pos_b is None:

return {'auc_a': auc_a, 'auc_b': auc_b, 'delta_auc': auc_a - auc_b,

'z_stat': None, 'p_value': None, 'se_diff': None,

'note': 'Single-class, cannot compute'}

V_a = np.zeros(n)

V_a[y_true == 1] = V_pos_a

V_a[y_true == 0] = V_neg_a

V_b = np.zeros(n)

V_b[y_true == 1] = V_pos_b

V_b[y_true == 0] = V_neg_b

var_a = np.var(V_a[y_true == 1], ddof=1) / n_pos + np.var(V_a[y_true == 0], ddof=1) / n_neg

var_b = np.var(V_b[y_true == 1], ddof=1) / n_pos + np.var(V_b[y_true == 0], ddof=1) / n_neg

cov_pos = np.cov(V_a[y_true == 1], V_b[y_true == 1], ddof=1)[0, 1] if n_pos > 1 else 0

cov_neg = np.cov(V_a[y_true == 0], V_b[y_true == 0], ddof=1)[0, 1] if n_neg > 1 else 0

cov_ab = cov_pos / n_pos + cov_neg / n_neg

se_diff = np.sqrt(max(0, var_a + var_b - 2 * cov_ab))

if se_diff < 1e-12:

return {'auc_a': auc_a, 'auc_b': auc_b, 'delta_auc': auc_a - auc_b,

'z_stat': 0, 'p_value': 1.0, 'se_diff': 0,

'note': 'SE ≈ 0'}

z_stat = (auc_a - auc_b) / se_diff

from scipy.stats import norm

p_value = 2 * norm.sf(abs(z_stat))

return {

'auc_a': round(auc_a, 4),

'auc_b': round(auc_b, 4),

'delta_auc': round(auc_a - auc_b, 4),

'se_diff': round(se_diff, 6),

'z_stat': round(z_stat, 4),

'p_value': round(p_value, 4),

'note': f'{"Significant" if p_value < 0.05 else "Not significant"} at α=0.05'

}

def run_delong_analysis():

print("=" * 60)

print("1. DeLong's Test: RF vs XGBoost")

print("=" * 60)

models_to_compare = {

'RandomForest': RandomForestClassifier(

n_estimators=500, max_depth=10, min_samples_leaf=20,

class_weight='balanced', random_state=RANDOM_STATE, n_jobs=-1

),

'XGBoost': xgb.XGBClassifier(

n_estimators=500, max_depth=5, learning_rate=0.03,

subsample=0.8, colsample_bytree=0.8,

eval_metric='logloss', random_state=RANDOM_STATE, verbosity=0, n_jobs=-1

),

}

all_results = []

for gender_key, gender_label in [('male', 'Male'), ('female', 'Female')]:

matrix_path = os.path.join(OUT_DIR, f'model_matrix_{gender_key}.pkl')

if not os.path.exists(matrix_path):

print(f" [{gender_label}] 模型矩阵缺失，跳过")

continue

data = joblib.load(matrix_path)

X = data['X']

y = data['y']

id_info = data['id_info']

groups = id_info['student_id'].values

if 'landmark_time' in X.columns:

X = X.drop(columns=['landmark_time'])

print(f"\n [{gender_label}] N={len(y)}, Events={int(y.sum())}")

sgkf = StratifiedGroupKFold(n_splits=N_CV_FOLDS, shuffle=True, random_state=RANDOM_STATE)

rf = models_to_compare['RandomForest']

rf_proba = cross_val_predict(rf, X, y, cv=sgkf, groups=groups,

method='predict_proba', n_jobs=-1)[:, 1]

xgb_model = models_to_compare['XGBoost']

neg_weight = (y == 0).sum() / (y == 1).sum()

xgb_model.set_params(scale_pos_weight=neg_weight)

xgb_proba = cross_val_predict(xgb_model, X, y, cv=sgkf, groups=groups,

method='predict_proba', n_jobs=-1)[:, 1]

result = delong_test(y, rf_proba, xgb_proba)

result['gender'] = gender_label

result['landmark'] = 'All'

print(f"	Overall: ΔAUC={result['delta_auc']}, z={result['z_stat']}, p={result['p_value']} → {result['note']}")

all_results.append(result)

for lm in [1, 2, 3]:

mask = id_info['landmark_time'].values == lm

if mask.sum() == 0 or y[mask].nunique() < 2:

continue

lm_result = delong_test(y[mask], rf_proba[mask], xgb_proba[mask])

lm_result['gender'] = gender_label

lm_result['landmark'] = f'Year {lm}'

print(f"	LM{lm}: ΔAUC={lm_result['delta_auc']}, z={lm_result['z_stat']}, p={lm_result['p_value']} → {lm_result['note']}")

all_results.append(lm_result)

df_delong = pd.DataFrame(all_results)

cols_order = ['gender', 'landmark', 'auc_a', 'auc_b', 'delta_auc',

'se_diff', 'z_stat', 'p_value', 'note']

df_delong = df_delong[[c for c in cols_order if c in df_delong.columns]]

df_delong.to_csv(os.path.join(TABLE_DIR, 'delong_test_results.csv'),

index=False, encoding='utf-8-sig')

print(f"\n [OK] delong_test_results.csv")

return df_delong

def compute_calibration_stats(y_true, y_pred_proba, n_bins=10):

from sklearn.linear_model import LogisticRegression

eps = 1e-12

proba_clipped = np.clip(y_pred_proba, eps, 1 - eps)

logit_pred = np.log(proba_clipped / (1 - proba_clipped))

X_logit = logit_pred.reshape(-1, 1)

cal_model = LogisticRegression(penalty=None, max_iter=5000)

cal_model.fit(X_logit, y_true)

cal_slope = cal_model.coef_[0][0]

cal_intercept = cal_model.intercept_[0]

from sklearn.calibration import calibration_curve

prob_true, prob_pred = calibration_curve(y_true, y_pred_proba, n_bins=n_bins)

e_max = np.max(np.abs(prob_true - prob_pred))

e_avg = np.mean(np.abs(prob_true - prob_pred))

brier = brier_score_loss(y_true, y_pred_proba)

return {

'calibration_slope': round(cal_slope, 4),

'calibration_intercept': round(cal_intercept, 4),

'brier_score': round(brier, 4),

'e_max': round(e_max, 4),

'e_avg': round(e_avg, 4),

}

def run_calibration_analysis():

print("\n" + "=" * 60)

print("2. Calibration Statistics (Slope & Intercept)")

print("=" * 60)

all_results = []

for gender_key, gender_label in [('male', 'Male'), ('female', 'Female')]:

pred_path = os.path.join(OUT_DIR, f'landmark_predictions_{gender_key}.csv')

if not os.path.exists(pred_path):

print(f" [{gender_label}] 预测数据缺失，跳过")

continue

preds = pd.read_csv(pred_path, encoding='utf-8-sig')

for lm in [1, 2, 3]:

mask = preds['landmark_time'] == lm

y_true = preds.loc[mask, 'y_true'].values

y_proba = preds.loc[mask, 'y_pred_proba'].values

if len(y_true) < 10 or len(np.unique(y_true)) < 2:

continue

stats = compute_calibration_stats(y_true, y_proba)

stats['gender'] = gender_label

stats['landmark'] = f'Year {lm}'

stats['n'] = len(y_true)

stats['n_events'] = int(y_true.sum())

stats['event_rate_pct'] = round(y_true.mean() * 100, 1)

print(f" [{gender_label}] LM{lm}: slope={stats['calibration_slope']:.3f}, "

f"intercept={stats['calibration_intercept']:.3f}, "

f"E_max={stats['e_max']:.3f}, Brier={stats['brier_score']:.3f}")

all_results.append(stats)

print(f"\n [{gender_label}] 按风险分层的校准...")

for lm in [1, 2, 3]:

mask = preds['landmark_time'] == lm

sub = preds[mask].copy()

if len(sub) < 30:

continue

sub['risk_group'] = pd.qcut(sub['y_pred_proba'], q=3,

labels=['Low', 'Medium', 'High'])

for group in ['Low', 'Medium', 'High']:

grp = sub[sub['risk_group'] == group]

obs_rate = grp['y_true'].mean() * 100

pred_rate = grp['y_pred_proba'].mean() * 100

row = {

'gender': gender_label,

'landmark': f'Year {lm}',

'risk_group': group,

'n': len(grp),

'n_events': int(grp['y_true'].sum()),

'observed_rate_pct': round(obs_rate, 1),

'predicted_rate_pct': round(pred_rate, 1),

'calibration_gap_pct': round(pred_rate - obs_rate, 1),

}

all_results.append(row)

df_cal = pd.DataFrame(all_results)

df_cal.to_csv(os.path.join(TABLE_DIR, 'calibration_stats.csv'),

index=False, encoding='utf-8-sig')

print(f"\n [OK] calibration_stats.csv")

return df_cal

def evaluate_logistic_model(X, y, groups, gender_label):

sgkf = StratifiedGroupKFold(n_splits=N_CV_FOLDS, shuffle=True, random_state=RANDOM_STATE)

lr = LogisticRegression(

penalty='l1', solver='saga', C=0.1,

max_iter=5000, random_state=RANDOM_STATE,

class_weight='balanced'

)

y_proba = cross_val_predict(

lr, X, y, cv=sgkf, groups=groups,

method='predict_proba', n_jobs=-1

)[:, 1]

y_pred = cross_val_predict(

lr, X, y, cv=sgkf, groups=groups,

method='predict', n_jobs=-1

)

rng = np.random.RandomState(RANDOM_STATE)

aucs = []

n = len(y)

for _ in range(N_BOOTSTRAP):

idx = rng.randint(0, n, n)

if y[idx].nunique() > 1:

aucs.append(roc_auc_score(y[idx], y_proba[idx]))

ci_low = np.percentile(aucs, 2.5)

ci_high = np.percentile(aucs, 97.5)

results = {

'gender': gender_label,

'model': 'Logistic_L1',

'auc_roc': round(roc_auc_score(y, y_proba), 4),

'auc_ci_low': round(ci_low, 4),

'auc_ci_high': round(ci_high, 4),

'brier_score': round(brier_score_loss(y, y_proba), 4),

'accuracy': round(accuracy_score(y, y_pred), 4),

'precision': round(precision_score(y, y_pred, zero_division=0), 4),

'recall': round(recall_score(y, y_pred, zero_division=0), 4),

'f1_score': round(f1_score(y, y_pred, zero_division=0), 4),

'n_samples': len(y),

'n_events': int(y.sum()),

'event_rate': round(y.mean() * 100, 1),

}

lm_results = []

for lm in [1, 2, 3]:

pass

return results, y_proba

def run_logistic_evaluation():

print("\n" + "=" * 60)

print("3. Logistic (Nomogram) Model CV Performance")

print("=" * 60)

all_results = []

for gender_key, gender_label in [('male', 'Male'), ('female', 'Female')]:

matrix_path = os.path.join(OUT_DIR, f'model_matrix_{gender_key}.pkl')

if not os.path.exists(matrix_path):

continue

data = joblib.load(matrix_path)

X = data['X']

y = data['y']

id_info = data['id_info']

groups = id_info['student_id'].values

if 'landmark_time' in X.columns:

X = X.drop(columns=['landmark_time'])

results, y_proba = evaluate_logistic_model(X, y, groups, gender_label)

print(f" [{gender_label}] Logistic AUC={results['auc_roc']} "

f"[95%CI: {results['auc_ci_low']}-{results['auc_ci_high']}], "

f"Brier={results['brier_score']}")

for lm in [1, 2, 3]:

mask = id_info['landmark_time'].values == lm

if mask.sum() == 0 or y[mask].nunique() < 2:

continue

lm_auc = roc_auc_score(y[mask], y_proba[mask])

lm_brier = brier_score_loss(y[mask], y_proba[mask])

cal_stats = compute_calibration_stats(y[mask], y_proba[mask])

lm_row = {

'gender': gender_label,

'landmark': f'Year {lm}',

'model': 'Logistic_L1',

'auc_roc': round(lm_auc, 4),

'brier_score': round(lm_brier, 4),

**cal_stats,

'n': int(mask.sum()),

'n_events': int(y[mask].sum()),

}

print(f"	LM{lm}: AUC={lm_auc:.4f}, Brier={lm_brier:.4f}, "

f"CalSlope={cal_stats['calibration_slope']:.3f}")

all_results.append(lm_row)

results['landmark'] = 'All'

all_results.append(results)

df_logistic = pd.DataFrame(all_results)

df_logistic.to_csv(os.path.join(TABLE_DIR, 'logistic_cv_performance.csv'),

index=False, encoding='utf-8-sig')

print(f"\n [OK] logistic_cv_performance.csv")

return df_logistic

def compute_trajectory_features_from_raw(df_long):

MEASURE_VARS = [

'total_score', 'bmi', 'vital_capacity_ml',

'endurance_run_sec', 'height_cm', 'weight_kg',

'run_50m_sec', 'long_jump_cm', 'sit_reach_cm', 'strength_zscore'

]

results = []

for sid, stu_data in df_long.groupby('student_id'):

stu_data = stu_data.sort_values('year_num')

for lm in [2, 3]:

lm_data = stu_data[stu_data['year_num'] <= lm]

if len(lm_data) < 2:

continue

current = lm_data[lm_data['year_num'] == lm]

baseline = lm_data[lm_data['year_num'] == 1]

if len(current) == 0 or len(baseline) == 0:

continue

for var in MEASURE_VARS:

if var not in lm_data.columns:

continue

vals = lm_data[['year_num', var]].dropna()

if len(vals) < 2:

continue

years = vals['year_num'].values

measures = vals[var].values

if years[-1] > years[0]:

slope = (measures[-1] - measures[0]) / (years[-1] - years[0])

else:

slope = np.nan

sd_val = measures.std() if len(measures) >= 2 else np.nan

delta_bl = current[var].values[0] - baseline[var].values[0] if pd.notna(current[var].values[0]) and pd.notna(baseline[var].values[0]) else np.nan

results.append({

'student_id': sid,

'landmark': lm,

'variable': var,

'slope_observed': slope,

'sd_observed': sd_val,

'delta_baseline_observed': delta_bl,

'n_obs_points': len(vals),

})

return pd.DataFrame(results)

def run_sensitivity_observed_vs_imputed():

print("\n" + "=" * 60)

print("4. Sensitivity: Observed-only vs Imputed Features")

print("=" * 60)

df_raw = pd.read_csv(os.path.join(OUT_DIR, 'data_long.csv'), encoding='utf-8-sig')

print(f" 加载原始数据: {len(df_raw)} 行, {df_raw['student_id'].nunique()} 人")

df_observed_features = compute_trajectory_features_from_raw(df_raw)

print(f" 仅观测值特征: {len(df_observed_features)} 行")

for gender_key, gender_label in [('male', 'Male'), ('female', 'Female')]:

lm_path = os.path.join(OUT_DIR, f'landmark_data_{gender_key}.csv')

if not os.path.exists(lm_path):

continue

df_lm = pd.read_csv(lm_path, encoding='utf-8-sig')

print(f"\n [{gender_label}] Landmark 数据集: {len(df_lm)} 行")

key_vars = ['total_score', 'bmi', 'vital_capacity_ml', 'endurance_run_sec']

for var in key_vars:

slope_col = f'{var}_slope'

sd_col = f'{var}_sd'

delta_col = f'{var}_delta_baseline'

for col in [slope_col, sd_col, delta_col]:

if col in df_lm.columns:

vals = df_lm[col].dropna()

print(f"	{col}: mean={vals.mean():.3f}, std={vals.std():.3f}, "

f"n_nonmissing={len(vals)}/{len(df_lm)}, "

f"min={vals.min():.3f}, max={vals.max():.3f}")

print(f"\n [{gender_label}] 仅观测值特征统计:")

df_obs_filtered = df_observed_features.merge(

df_lm[['student_id']].drop_duplicates(), on='student_id', how='inner'

)

for var in key_vars:

sub = df_obs_filtered[df_obs_filtered['variable'] == var]

for metric in ['slope_observed', 'sd_observed', 'delta_baseline_observed']:

vals = sub[metric].dropna()

if len(vals) > 0:

print(f"	{var}_{metric}: mean={vals.mean():.3f}, std={vals.std():.3f}, "

f"n={len(vals)}, min={vals.min():.3f}, max={vals.max():.3f}")

df_observed_features.to_csv(

os.path.join(TABLE_DIR, 'sensitivity_observed_features.csv'),

index=False, encoding='utf-8-sig'

)

print(f"\n [OK] sensitivity_observed_features.csv")

return df_observed_features

def main():

print("=" * 60)

print("返修分析: DeLong + Calibration + Logistic + Sensitivity")

print("=" * 60)

df_delong = run_delong_analysis()

df_cal = run_calibration_analysis()

df_logistic = run_logistic_evaluation()

df_sens = run_sensitivity_observed_vs_imputed()

print("\n" + "=" * 60)

print("全部返修分析完成")

print("=" * 60)

print(f" 输出: {TABLE_DIR}/")

print(f"	delong_test_results.csv")

print(f"	calibration_stats.csv")

print(f"	logistic_cv_performance.csv")

print(f"	sensitivity_observed_features.csv")

if __name__ == '__main__':

main()
